# Characterization of Xi-class mycothiol S-transferase from *Corynebacterium glutamicum* and its protective effects in oxidative stress

**DOI:** 10.1186/s12934-019-1232-8

**Published:** 2019-10-26

**Authors:** Meiru Si, Chengchuan Che, Guanxi Li, Xiaona Li, Zhijin Gong, Jinfeng Liu, Ge Yang, Can Chen

**Affiliations:** 1 College of Life Sciences, Qufu Normal University, Qufu, 273165 Shandong China; 2 College of Life Science and Agronomy, Zhoukou Normal University, Zhoukou, 466001 Henan China

**Keywords:** Glutathione transferase Xi, Mycothiolyl-hydroquinone reductase, Mycothiolyl-acetophenone reductase, Redox regulation, *Corynebacterium glutamicum*, Oxidative stress

## Abstract

**Background:**

Oxidative stress caused by inevitable hostile conditions during fermentative process was the most serious threat to the survival of the well-known industrial microorganism *Corynebacterium glutamicum.* To survive, *C. glutamicum* developed several antioxidant defenses including millimolar concentrations of mycothiol (MSH) and protective enzymes. Glutathione (GSH) S-transferases (GSTs) with essentially defensive role in oxidative stress have been well defined in numerous microorganisms, while their physiological and biochemical functions remained elusive in *C. glutamicum* thus far.

**Results:**

In the present study, we described protein NCgl1216 belonging to a novel MSH S-transferase Xi class (MstX), considered as the equivalent of GST Xi class (GSTX). MstX had a characteristic conserved catalytic motif (Cys-Pro-Trp-Ala, C-P-W-A). MstX was active as thiol transferase, dehydroascorbate reductase, mycothiolyl-hydroquinone reductase and MSH peroxidase, while it showed null activity toward canonical GSTs substrate as 1-chloro-2,4-dinitrobenzene (CDNB) and GST Omega’s specific substance glutathionyl-acetophenones, indicating MstX had some biochemical characteristics related with mycoredoxin (Mrx). Site-directed mutagenesis showed that, among the two cysteine residues of the molecule, only the residue at position 67 was required for the activity. Moreover, the residues adjacent to the active Cys67 were also important for activity. These results indicated that the thiol transferase of MstX operated through a monothiol mechanism. In addition, we found MstX played important role in various stress resistance. The lack of *C. glutamicum mstX* gene resulted in significant growth inhibition and increased sensitivity under adverse stress condition. The *mstX* expression was induced by stress.

**Conclusion:**

*Corynebacterium glutamicum* MstX might be critically involved in response to oxidative conditions, thereby giving new insight in how *C. glutamicum* survived oxidative stressful conditions.

## Background

Glutathione S-transferases (GSTs), the inducible phase II enzymes, comprised a superfamily of multifunctional enzymes widely distributed among living organism [1]. GSTs played crucial roles in several cellular functions, and had various toxicants acceptance, including drugs, xenobiotics, reactive oxygen species (ROS) [1]. GSTs adopted a conserved fold composed of two domains: an N-terminal domain with a conserved thioredoxin-like motif containing the glutathione (GSH) binding pocket (G-site), and a unique α-helical C-terminal domain (H-site) containing the hydrophobic site of substrate-binding GSH [2]. Based on a characteristic conserved amino acid residue, GSTs were divided into two classes: Ser/Tyr-GSTs and Cys-GSTs. Most GSTs, belonging to Ser/Tyr-GSTs class, harbored a catalytic serine or tyrosine residue at the G-site in N-terminus [3]. The Ser or Tyr residue was responsible for the nucleophilic attack on substrate, making GSTs conjugate GSH to the electrophilic groups of a variety of the hydrophobic molecules. Thus, Ser/Tyr-GSTs displayed glutathionylation activity [3]. However, Cys-GSTs contained a catalytic cysteine residue at the G-site in N-terminus, catalyzing GSH removal from specific substrates and was simultaneously oxidized through the formation of an enzyme-GSH mixed disulfide with the concomitant reduction of a co-substrate. Thus, Cys-GSTs mainly exhibited deglutathionylation activity [4]. Ubiquitous glutathionylation function of Ser/Tyr-GSTs not only could neutralize free radicals and active components, but also cause the formation of GSH conjugates. Many studies showed that GSH conjugates were more soluble, easier to be excreted from cells, and less toxic than the original compounds [5]. The deglutathionylation function of Cys-GSTs facilitated the proper folding of proteins. Ser/Tyr-GSTs were usually dimeric, while Cys-GSTs exhibited more structural diversity, including monomer, the canonical dimer and atypical dimer. Although Cys-GSTs contained less members than Ser/Tyr-GSTs, Cys-GSTs also represented important GST class. Cys-GSTs were further classified into about 6 subclasses on biochemical characterization so far, including Xi [6], Omega [7], Beta [8], CLIC [9], dehydroascorbate reductases [10], and Lambda [4].

The GSTs Xi class (GSTXs) had been well studied in bacteria [11, 12], fungi [13], *P. chrysosporium* [6], and plants [14], and constituted a new phylogenetic group of GSTs [6]. In fact, GSTXs were initially identified as the GST omegas subclass I (GSTOIs), once named *S*-glutathionyl-(chloro) hydroquinone reductase (GHR). Hereafter, Meux and co-worker believed, on the basis of sequence analysis and biochemical and structural differences from canonical GSTOs, that GHRs of the GSTOIs should be considered as a new class of GSTs, reclassifying them as GSTXs [6]. In summary, the activities associated to GSTXs were: (i) GSH-dependent thiol transferase activity, being attributable to its structural and functional similarity to glutaredoxins (Grxs), but low or lacking activity toward classical GSTs substrate as 1-chloro-2,4-dinitrobenzene (CDNB) [15]. (ii) Catalyses of several reductive reactions in cellular biochemistry, including dehydroascorbate (DHA) and monomethylarsonic acid (MMA) [15]. As a DHA reductase, GSTXs reduced DHA for ascorbic acid (AA). AA played a major role in scavenging free radical and ROS [16]. Thus, GSTXs played an important protective role in the response to oxidative stress. The above two catalytic functions of GSTXs, usually also existing in GSTOs, rarely existed in members of the Ser/Tyr-GST. (iii) Specific reduction of *S*-glutathionyl-(chloro)hydroquinone (GH), and such activity was not detected for the other GSTOs [6]. However, unlike GSTOs, GSTXs did not have the activity of reducing glutathionyl-acetophenones (which was once thought to be specific to GSTOs), although more recently Schwartz et al. [15] found that the GSTX3 from the white rot *Trametes versicolor* (TvGSTX3) had glutathionyl-acetophenone and GH reductase activities. That is to say that TvGSTX3 reduced both Xi and Omega substrates [15]. However, nothing was known about the universality and exact molecular mechanism of the catalytic duality of GSTXs. Thus, an in-depth analysis of the catalytic mechanism of GSTXs was a subject of active investigation.

*Corynebacterium glutamicum* not only was a well-known industrial microorganism widely used for the amino acids and nucleotides production, but also was an excellent model organism to study the physiology of its pathogenic relatives such as *Mycobacterium tuberculosis*, *C. jeikeium* and *C. diphtheriae* [17]. During fermentative process, *C. glutamicum* inevitably encountered a series of unfavorable circumstances including high osmotic pressure, antibiotics, nutrient starvation, low pH, and/or oxidation [18]. However, *C. glutamicum* robustly survived the adverse stresses of the fermentative process using several antioxidant defenses, such as millimolar concentrations of low molecular weight (LMW) thiol mycothiol (MSH) and antioxidant enzymes [19, 20]. Sufficient evidences showed that MSH had the equivalent functions with GSH in bacteria [19, 20]. MSH played an important role in the defence against several external stresses including oxidative stress, alkylating agents and antibiotics [19, 20]. Also, our previous studies showed that many antibiotics and electrophilic compounds were conjugated to MSH and then were detoxified by MSH *S*-conjugate amidase (Mca) [21]; many antioxidant proteins, including MSH peroxidase MPx, methionine sulfoxide reductase MsrA, and peroxiredoxin Prx, were discovered as involving in mycothiolation/demycothiolation when detoxifying various peroxides [22–24]. These studies suggested that the putative MSH S-transferase (MST) catalyzing MSH-dependent detoxification reactions existed in *C. glutamicum.* Newton et al. identified MSMEG_0887 from *M. smegmatis* and Rv0443 from *M. tuberculosis,* the member of the DinB superfamily, as MSTs, while an MSH-dependent protein of the DinB superfamily that was homology with *M. smegmatis* MSMEG_0887 showed maleylpyruvate isomerase activity in *C. glutamicum* [25, 26]. Interestingly, previous study identified a putative GSH S-transferase (GST) (NCgl1216) negatively regulated by a hydrogen peroxide-sensitive MarR (multiple antibiotic resistance regulator)-type sensor RosR (regulator of oxidative stress response) using DNA microarrays in *C. glutamicum* [27]. This study suggested that NCgl1216 might be the MSH-dependent MST. Therefore, we sought to explore the physiological and biochemical function of NCgl1216 of *C. glutamicum*.

## Methods

### Strains and culture conditions

The bacterial strains and plasmids that were in this study listed in Additional file 1: Table S1. Luria–Bertani (LB) broth and LB agar plates were used for growing *Escherichia coli* or *C. glutamicum. E. coli* and *C. glutamicum* were cultivated at 37 °C and 30 °C under vigorous agitation (220 rpm) as previously reported, respectively [26]. 0.5 M sorbitol-containing brain–heart broth medium (BHIS) was used for producing and maintaining mutant of a gene in *C. glutamicum* [26]. To create a *mstX* (*ncgl1216*) gene deletion in *C. glutamicum* RES167 parental strain, the pK18*mobsacB*-Δ*mstX* plasmids were transformed into *C. glutamicum* WT through electroporation according to the method of Shen et al. and then integrated into the chromosome of *C. glutamicum* through homologous recombination to execute single crossover [26]. The transconjugants were selected on LB agar plate in the presence of 40 µg ml^−1^ nalidixic acid (NAL) and 25 µg ml^−1^ kanamycin (KAN). Counter-selection for markerless in-frame deletion was carried out on 40 µg ml^−1^ NAL and 20% (W/V) sucrose-containing LB agar plates [28]. Strains growing on this plate were detected for KAN sensitivity (KAN^S^) by parallel picking on LB plates in the presence of 40 µg ml^−1^ NAL and 25 µg ml^−1^ KAN or 40 µg ml^−1^ NAL and 20% sucrose. Sucrose-resistant and KAN-sensitive strains were detected for in-frame deletion by PCR using the DmstX-F1/DmstX-R2 primer pair (Additional file 1: Table S2) and verified by DNA sequencing. To create complementary strains, the pXMJ19-*mstX* plasmids were transformed into Δ*mstX* mutants by electroporation [29]. 0.5 mM isopropyl β-d-1-thiogalactopyranoside (IPTG) was added into medium to induce the expression of *mstX* gene on the pXMJ19-*mstX* in complementary strains. For constructing chromosomal fusion reporter strains, the plasmid pK18*mobsacB*-*P*_*mstX*_*::lacZY* was transformed into relevant *C. glutamicum* strains by electroporation. The chromosomal pK18*mobsacB*-*P*_*mstX*_*::lacZY* fusion reporter strain was selected on LB agar plates added with 25 µg ml^−1^ KAN and 40 µg ml^−1^ NAL. All chemicals were of analytical reagent grade purity or higher. Antibiotics were added at the following concentrations: KAN, 50 µg ml^−1^ for *E. coli* and 25 µg ml^−1^ for *C. glutamicum*; NAL, 40 µg ml^−1^ for *C. glutamicum*; chloramphenicol (CHL), 20 µg ml^−1^ for *E. coli* and 10 µg ml^−1^ for *C. glutamicum*.

### Plasmid construction

Primers in this study were listed in Additional file 1: Table S2. Primers for the amplification of genes and real-time reverse transcription-PCR (RT-PCR) were synthesized at Sangon Biotech Co., Ltd. (Shanghai, China). For giving expression plasmids, the *mstX* gene (*ncgl1216*) region of *C. glutamicum* was amplified with primers OmstX-F and OmstX-R from genomic DNA of *C. glutamicum* RES167 by PCR, and then the resulting fragments cut with *Bam*HI and *Hin*dIII enzymes were cloned into appropriately digested pET28a to give plasmid pET28a-*mstX.*

To create the Δ*mstX* in-frame deletion mutant, the suicide plasmid pK18*mobsacB*-Δ*mstX* was constructed by overlap PCR [28, 30]. First, based on DNA sequences of *mstX* gene and its adjacent regions, two oligonucleotide primer pairs namely DmstX-F1/DmstX-R1 and DmstX-F2/DmstX-R2 listed in Additional file 1: Table S2 were made (Sangon Biotech Co., Ltd., Shanghai, China). Primer pair DmstX-F1/DmstX-R1 was used to amplify the *mstX*’s upstream 852 bp fragment; while primer pair DmstX-F2/DmstX-R2 was used to amplify the *mstX*’s downstream 816 bp fragment. The upstream and downstream fragments were fused together by overlap PCR with the primer pair DmstX-F1/DmstX-R2. The resulting PCR products were cut with *Eco*RI and *Bam*HI and then clouded into similar sites of pK18*mobsacB* vector to generate plasmid pK18*mobsacB*-Δ*mstX*.

To produce pXMJ19-*mstX*, primer pair CmstX-F/CmstX-R was designed to amplify the DNA fragments of open reading frames region of *mstX* gene from *C. glutamicum* genomic DNA. The amplified DNA fragments were cut with *Bam*HI and *Eco*RI and then cloned into pXMJ19 vector between *Bam*HI and *Eco*RI sites.

To obtain the DNA fragment of mutant *mstX:C67S*, site-directed mutagenesis was carried out by two rounds of PCR [31]. Briefly, two oligonucleotide primer pairs namely DmstX-F1/mstX-C67S-R and mstX-C67S-F/DmstX-R2 listed in Additional file 1: Table S2 were designed and synthesized. In the first round of PCR, primer pair DmstX-F1/mstX-C67S-R was used to amplify the 5′ prime region of *mstX* (Fragment I); while primer pair mstX-C67S-F/DmstX-R2 was used to amplify the 3′ prime region of *mstX* (Fragment II). The second round of PCR was performed by using OmstX-F/OmstX-R as primers and fragment I and fragment II as templates to get the *mstX:C67S* fragment. The *Bam*HI and *Hin*dIII cut *mstX:C67S* DNA fragments were cloned in pET28a plasmid digested with similar enzymes to create plasmid pET28a-*mstX:C67S*. The *mstX:C67*G, *mstX:C67*A, *mstX:C67Y*, *mstX:P68G*, *mstX:W69F*, *mstX:A70S*, *mstX:V192A*, *mstX:N193A*, *mstX:G235L*, *mstX:I238A*, *mstX:T239A*, *mstX:D242G*, *mstX:I243S*, *mstX:T248A*, *mstX:R251A*, *mstX:C262G,* and *mstX:C262S* fragments were obtained using a similar procedure as described above and cloned into pET28a to result in corresponding plasmid derivatives.

For getting the *lacZY* fusion reporter vector pK18*mobsacB*-*P*_*mstX*_*::lacZY*, the fusion of *mstX* promoter to the *lacZY* reporter gene by overlap PCR was created. First, two oligonucleotide primer pairs namely P_*mstX*_-F/P_*mstX*_-R and lacZY-F/lacZY-R were designed in the first round of PCR to amplify the 200-bp *mstX* promoter DNA fragments (corresponding to nucleotides + 12 to − 188 relative to the translational start codon (ATG) of *mstX* gene) and the *lacZY* DNA fragments, respectively. Second, P_*mstX*_-F/lacZY-R as primers and the first round PCR products as templates were used to carry out the second round of PCR, and the resulting fragments cut with *Sma*I and *Bam*HI were inserted into pK18*mobsacB* between *Sma*I and *Bam*HI sites to get the pK18*mobsacB*-*P*_*mstX*_*::lacZY* fusion construct [28, 29]. The fidelity of all constructs was confirmed by DNA sequencing (Sangon Biotech, Shanghai, China).

### Plasmid construction overexpression and purification of recombinant protein

The overexpression of His_6_-MstX protein in *E. coli* BL21 (DE3) cells harboring pET28a derivatives plasmids and purification of the overexpressed protein with the His · Bind Ni^2+^-nitrilotriacetate (Ni-NTA) resin (Novagen, Madison, WI) were carried out as described previously [29]. Eluted recombinant His_6_-MstX proteins were concentrated and loaded onto a Superdex-75 10/300 gel filtration column (GE Healthcare, Piscataway, NJ) with a running condition of 10 mM Tris (pH 7.4), 100 mM NaCl, and 5 mM β-mercaptoethanol. Cleavage of the His_6_ tag was performed by adding 10 units of Enterokinase-Max (Invitrogen, Karlruhe, Germany) and incubation at 4 °C overnight to conduct subsequent enzyme activity analysis. Ni-NTA agarose was used to remove the cleaved tag and uncleaved protein from the tag-free protein. All enzymes were purchased from Sigma-Aldrich (St. Louis, MO). The resulting His_6_-tag-free proteins were dialyzed against PBS at 4 °C and concentrated for further experiments [> 95% purity as estimated by sodium dodecylsulphate polyacrylamide gel electrophoresis (SDS-PAGE)].

### Sensitivity assays for oxidants, antibiotics, alkylating agents, and heavy metals

The minimum inhibitory concentration (MIC) assay was performed for oxidants according to Rawat et al. [32]. Briefly, after oxidants were serially diluted (0.5×) in LB medium (1 ml), cells grown to the stationary phase were inoculated with 1% inoculum. After 1 to 2 days of cultivation at 30 °C, the tubes were checked for growth. The bacteriostasis growth curve assay was used to determine the sensitivity of *C. glutamicum* to heavy metal stress. LB-grown strains (50 μl) were transferred to LB broth (5 ml), with and without addition of various concentrations of heavy metals (Cd^2+^, Ni^2+^ and Cu^2+^) and incubated at 30 °C. The cellular growth was monitored turbidimetrically after 24 h. Antibiotics and alkylating agents experiments were performed according to Liu et al. [20] with minor modifications as follows: Overnight cultures of *C. glutamicum* RES167 strain were diluted in 1:100 in fresh LB medium with indicated concentration of various antibiotics or alkylating agents and cultivated for over 24 h with shaking at 30 °C [20]. Cellular growth was monitored by determining optical density at 600 nm at indicated time points. All assays were performed in triplicate.

### Quantitative analysis of sulfhydryl groups

Free sulfhydryl groups in MstX WT and its variants were measured using 5,5′-dithio-bis(2-nitrobenzoic acid) (DTNB) [33]. After 10 μM proteins were treated with 200 μM H_2_O_2_ and 50 mM dithiothreitol (DTT) at room temperature for 30 min, respectively, followed by removing residual DTT or H_2_O_2_ with a PD10 desalting column (GE Healthcare, Piscataway, NJ). The resulting proteins (10 μM) were added to 2 mM DTNB in 50 mM Tris–HCl buffer (pH8.0) and the absorbance at 412 nm was measured against a 2 mM DTNB solution as the reference. The amounts of reactive sulfhydryl groups were measured using the molar absorption coefficient of TNB at 412 nm (*ε*_412_) of 13,600 M^−1^ cm^−1^ [34].

### In vitro state of MstX under stress

The redox states of MstX WT and its variants (10 μM) were analyzed by incubating the proteins with 200 μM H_2_O_2_, 600 μM MSH, 200 μM H_2_O_2_ + 600 μM MSH, or 200 μM H_2_O_2_ + 600 μM MSH + 50 mM DTT for 30 min before separating on nonreducing 15% SDS-PAGE. For nonreducing conditions, DTT and β-mercaptoethanol were omitted from the loading buffer. All the samples were boiled for 5 min prior to electrophoresis.

### Detection of S-mycothiolated MstX:C262S in vitro

For in vitro test, S-mycothiolated MstX:C262S (MstX:C262S-SSM, the mixed disulphide between MSH and MstX:C262S-SH) was produced according to the method of Chi et al. [35]. MSH was purified from *C. glutamicum* RES167 as described previously [29]. MstX:C262S (50 μM) was incubated with excess of reduced MSH (6 M) prior to the addition of 2 mM H_2_O_2_. After 30 min incubation, excess MSH and H_2_O_2_ were removed by ultrafiltration. The sample was loaded on Ni-NTA His·Bind resin-containing column (Novagen, Wisconsin, USA) and S-mycothiolated MstX:C262S that could not combine with resin was directly collected. To obtain a pure sample, collected solution containing S-mycothiolated MstX:C262S was purified on a superdex75HR column equilibrated with 50 mM HEPES/NaOH pH 8.0, 150 mM NaCl. Pure S-mycothiolated MstX:C262S were confirmed by Matrix-Assisted Laser Desorption/Ionization Time of Flight Mass Spectrometry (MALDI-TOF MS).

### The thioredoxin (Trx)/thioredoxin reductase (TrxR) and mycothione reductase (Mtr)/MSH electron transfer assays

The reduction of MstX:C262S-SSM was performed by monitoring the decrease in absorbance at 340 nm arising from NADPH oxidation. The assay mixtures contained 50 mM HEPES/NaOH pH 8.0 buffer, 500 μM NADPH, 50 μM MstX:C262S-SSM, and a reduced Trx-generating system [4 μM TrxR and 40 μM Trx], or MSH system [4 μM Mtr and 500 μM MSH] as the possible electron donors. All concentrations were calculated taking into account the subsequent addition of MstX:C262S-SSM. After the mixtures were incubated for 30 min at 37 °C, the reactions were started by adding different concentration of MstX:C262S-SSM. For each reaction mixture with a control well, MstX:C262S-SSM deleted from the reaction mixture was added. Three independent experiments were performed at each concentration. The *k*_cat_ and *k*_m_ values of MstX:C262S-SSM for MSH were obtained from a non-linear fit with the Michaelis–Menten equation using the program GraphPad Prism 5.

### Enzymatic activity assay and enzyme kinetics

Activities with hydroxyethyl disulphide (HED) and DHA as substrates were determined through the reduction of the mixed disulphide formed between HED and MSH or DHA and MSH, as described previously [36]. The HED and DHA assay for GSH was modified for MSH [37]. The kinetic parameters were determined in the presence of varying concentration of DHA or HED (0.05–10 mM) with a saturating concentration of 100 mM MSH, while that for MSH (0.1–10 mM) was determined in the presence of a saturating concentration of 100 mM HED or DHA. The enzyme reactions were measured in 50 mM potassium phosphate buffer (pH 7.6), 300 μM NADPH, 1 μM His tag-free MstX, and 5 μM Mtr. The assay was performed at 25 °C and the absorption monitored at 340 nm. The activity was determined after subtracting the spontaneous reduction rate observed in the absence of MstX, and the number of micromoles of NADPH oxidized per second per micromole of enzyme (i.e. turnover number, s^−1^) was calculated using the molar absorption coefficient of NADPH at 340 nm (*ε*_340_) of 6220 M^−1^ cm^−1^. Three independent experiments were performed at each substrate concentration. The *k*_cat_ and *k*_m_ values of MstX for MSH, HED or DHA substrates were obtained from a non-linear fit with the Michaelis–Menten equation using the program GraphPad Prism 5.

The reductase activities for mycothiolyl-phenylacetophenone (MS-PAP) and mycothiolyl-menadione (MS-MEN), were tested as described previously [38]. The appearance of the expected products (phenylacetophenone and menadione) was followed by reverse phase chromatography (4.6 × 250 mm Ecosil C18-AQ/C18-AQ PLUS column No. AJ1205-1546, Japan, Tokyo). The assays were carried out as developed previously [4]. Catalytic parameters were determined using varying substrate concentrations (0.05–10 mM MS-PAP, or 0.5–10 mM MS-MEN) at saturating MSH concentration (100 mM), respectively, while that for MSH (0–20 mM) was determined in the presence of a saturating concentration of 50 mM MS-MEN. The *k*_cat_ and *k*_m_ values were obtained from a non-linear fit with the Michaelis–Menten equation using the program GraphPad Prism 5.

GST activities were spectrophotometrically determined for different substrates [39].

Peroxidase activity assays were performed by monitoring the decrease in absorbance at 340 nm arising from NADPH oxidation [40]. The catalytic properties of MstX were determined using a reduced system (Mtr and 500 μM MSH) as the possible electron donors. These assays were carried out in a total volume of 500 μl containing 50 mM Tris–HCl buffer (pH 7.5), 1 mM EDTA, 250 μM NADPH, 1 μM MstX, 500 μM MSH, 4 μΜ Mtr. The reactions were started by addition of peroxide substrates following 5 min of preincubation. The catalytic parameters of MstX for peroxides have been obtained by varying peroxide concentration at saturating concentrations of the other substrate (between 0 and 2000 µM for peroxides). NADPH oxidation was monitored as *A*_340_. The activity was determined after subtracting the spontaneous reduction rate observed in the absence of MstX, and the number of micromoles of NADPH oxidized per second per micromole of enzyme (i.e. turnover number, s^−1^) was calculated using the molar absorption coefficient of NADPH at 340 nm (*ε*_340_) of 6220 M^−1^ cm^−1^. Three independent experiments were performed at each substrate concentration. The *k*_cat_ and *k*_m_ values were obtained from a non-linear fit with the Michaelis–Menten equation using the program GraphPad Prism 5.

The Ferrous Oxidation of Xylenol orange (FOX) assay was performed as described [41].

### β-galactosidase assay

β-Galactosidase activities were detected using *o*-nitrophenyl-β-d-galactopyranoside (ONPG) as the substrate [42].

### Quantitative RT-PCR analysis

Quantitative RT-PCR analysis (7500 Fast Real-Time PCR; Applied Biosystems, Foster City, CA) was performed as described previously [30]. The primers used were listed in Additional file 1: Table S2. To obtain standardization of results, the relative abundance of 16S rRNA was used as the internal standard.

### Statistical analysis

GraphPad Prism Software was used to carry out statistical analyses (GraphPad Software, San Diego California USA).

## Results and discussion

### Involvement of MstX in oxidative resistance

The *ncgl1216* gene of *C. glutamicum* RES167 encoded a protein of 359 amino acids (mass, 40,619 Da), which has been annotated as a putative protein of GSH S-transferase (GST) family. Amino acid sequence alignment showed that NCgl1216 had very high similarity with representative Archaea and Gram-positive GSTs Xi class containing a Cys-Pro-Trp-Ala (C-P-W-A) typical active site motif, the hydrophonic co-substrate, the N-capping box (TXXD), and the hydrophodic stable motif (Additional file 1: Figure S1). Previous works suggested the GSTs Xi class were functionally related to Grxs [15], and Grx provided defense against oxidants and other stresses [43]. However, the role of NCgl1216 protein has not been studied so far. To get clues on the function of NCgl1216 in defending against condition stress, we determined the phenotype of a *ncgl1216* knockout in *C. glutamicum* to various agents by the MIC and growth curve inhibition assay. As shown in Table 1, the MIC of the tested oxidizing agents [hydrogen peroxide (H_2_O_2_), menadione (MEN), cumene hydroperoxide (CHP) and diamide] for Δ*ncgl1216* mutant (strains lacking *ncgl1216* gene contained empty pXMJ19) was lower than that for *C. glutamicum* parental strain containing the high copy number of empty plasmid pXMJ19 (the strains were named WT). For all four oxidants, the sensitivity of the Δ*ncgl1216* mutant was rescued by introduction of a plasmid expressing wild type *ncgl1216* gene, indicating that the lack of *ncgl1216* gene was strongly linked to the increased sensitivity of the Δ*ncgl1216* mutant (Table 1).

**Table 1:** The minimum inhibitory concentrations (MICs) of various oxidants for *C*. *glutamicum* strains

Oxidants	MIC (mM)		
	WT	Δ*mstX*	Δ*mstX*^+^
H_2_O_2_	120–100	60	100–80
Diamide	6.4–8	3.2–1.6	6.4
CHP	1.1	0.55–0.275	1.1
MEN	3.6	1.8–0.9	3.6

*H*_*2*_*O*_*2*_ hydrogen peroxide, *CHP* cumene hydroperoxide, *MEN* menadione

**Fig. 1:**
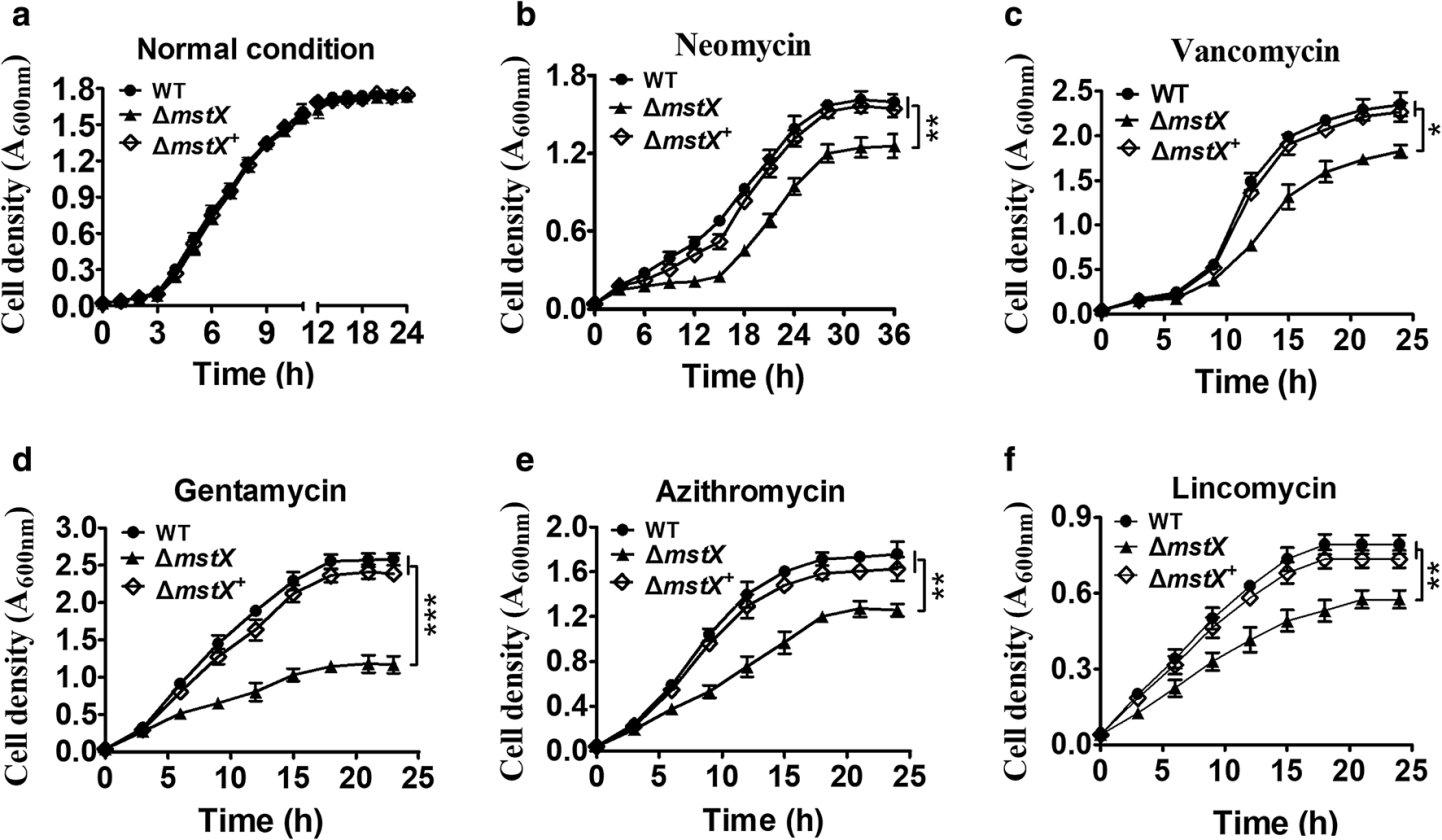
Growth of *C. glutamicum* RES167 parental strain containing the high copy number of empty plasmid pXMJ19 (WT), Δ*mstX* mutant (strains lacking *mstX* gene contained empty pXMJ19) and the complementary strains Δ*mstX*^+^ (Δ*mstX* mutant was complemented with plasmids carrying the wild-type *mstX* gene) in LB broth with 0.25 μg ml^−1^ neomycin (**b**), 0.2 μg ml^−1^ vancomycin (**c**), 0.05 μg ml^−1^ gentamycin (**d**), 24 μg ml^−1^ azithromycin (**e**), and 110 μg ml^−1^ Lincomycin (**f**). Bacterial growth was monitored by the optical density at 600 nm for up to 24 h after addition of the same cell density of exponentially grown culture in aerobic condition. Growth of the three strains in LB broth without antibiotic was used as control (**a**). Mean values with standard deviations (error bars) from at least three repeats were shown. ***P ≤ 0.01; **P ≤ 0.01; *P ≤ 0.05

**Fig. 2:**
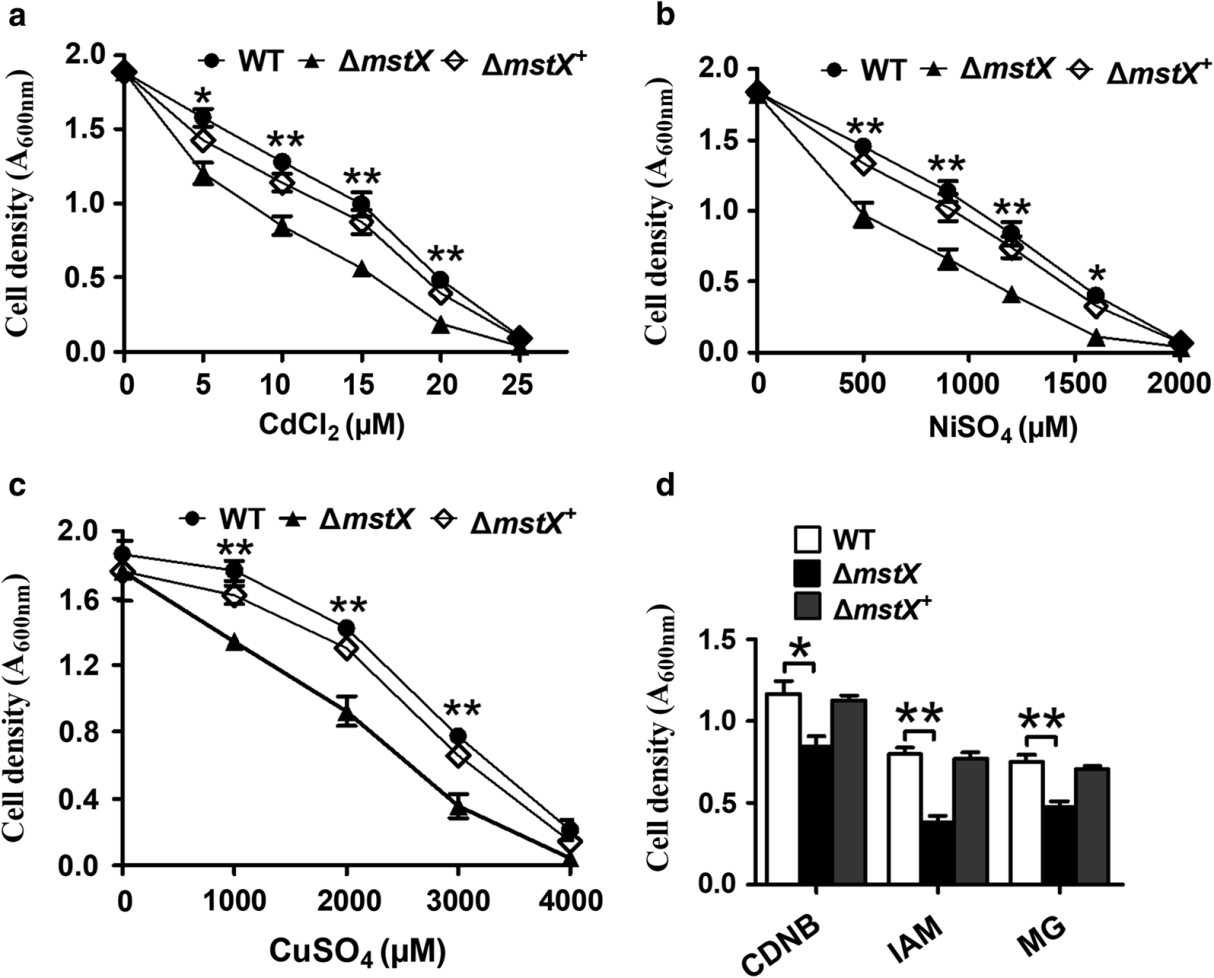
Effect of deletion of *mstX* gene on heavy metal and alkylating agent resistance. **a**–**c** Dose–response curves for the *C. glutamicum* WT, Δ*mstX* and Δ*mstX*^+^ strains over 24 h at 30 °C in LB medium containing increasing concentrations of CdCl_2_ (**a**), NiSO_4_ (**b**), and CuSO_4_ (**c**) were recorded. **d** The growth of the *C. glutamicum* WT, Δ*mstX* and Δ*mstX*^+^ strains after 24 h at 30 °C in LB medium containing CDNB (7.5 mM), IAM (5 mM), and MG (2.5 mM) were recorded. Mean values with standard deviations (error bars) from at least three repeats were shown. **P ≤ 0.01; *P ≤ 0.05

Antibiotics, alkylating agents and heavy metals could contribute to oxidative cellular conditions through a common mechanism: inducing ROS formation [44–46]. To examine the impact of NCgl1216 on resistance to antibiotics, alkylating agents and heavy metals, the strain growth in LB broth media supplemented with different agents was detected. All the strains tested had nearly identical levels of growth in the LB broth media, while WT and the complementary strain ∆*ncgl1216*^+^ (Δ*ncgl1216* mutant was complemented with plasmids carrying the wild-type *ncgl1216* gene) obviously grew better than the Δ*ncgl1216* mutant under neomycin, vancomycin, gentamycin, lincomycin, azithromycin, iodoacetamide (IAM), 2,4-dinitrochlorobenzene (CDNB), methylglyoxal (MG), CdCl_2_, NiSO_4_, and CuSO_4_ challenge (Figs. 1, 2). In short, these results confirmed that NCgl1216 had an influence on the *C. glutamicum* oxidative stress response. Thus, we named NCgl1216 as MstX (mycothiol S-transferase Xi class).

### In vitro MstX state upon oxidation

To investigate the molecular and biochemical property of MstX, we cloned the open-reading frame of MstX into the pET28a plasmid and expressed the recombinant proteins in *E. coli* (BL21). Recombinant MstX was produced as a fusion with a 6 × His tag and purified with a Ni^2+^-affinity resin. The molecular mass of recombinant His_6_-MstX was estimated to be ~ 45 kDa by nonreducing SDS-PAGE gel (Additional file 1: Figure S2a), in close agreement with the sum of its native molecular weight of 39.5 kDa and His_6_ (about 5 kDa). The result showed that MstX was monomeric, different from the reported GSTXs, mainly in the form of dimer [15].

Amino acid sequence alignment showed that MstX had two cysteine residues (at positions 67 and 262, as shown in Additional file 1: Figure S1). To reveal the state of Cys in MstX under oxidant treatment, the two variants, C67S and C262S, with each Cys mutated to Ser, respectively, were prepared, and the analytical methods of nonreducing SDS-PAGE gel and DTNB were used. H_2_O_2_-treated MstX WT still appeared as monomer on nonreducing SDS-PAGE gel, suggesting that MstX WT was functionally monomeric and did not form disulfide bond-containing dimer (Additional file 1: Figure S2b). But what’s interesting was that MstX WT treated with H_2_O_2_ and MSH at the same time migrated more slowly than the forms treated only with MSH or H_2_O_2_. Importantly, MstX WT treated with H_2_O_2_ and MSH at the same time could be reduced by adding excessive DTT, indicating that these bands were reversed (Additional file 1: Figure S2b). Similarly, MstX:C262S treated with H_2_O_2_ and MSH at the same time migrated more slowly than the forms treated only with MSH or H_2_O_2_. However, the migration status of MstX:C67S treated with H_2_O_2_ and MSH simultaneously was similar to that of MstX:C67S under DTT treatment. Since MSH can reversibly modify sulfhydryl groups of some protein by combination, the apparent delay of electrophoretic mobility would occur in proportion to the number of free thiol groups in proteins. Therefore, according to our results, we speculated that S-mycothiolation was generated on Cys67 of MstX. DTNB assay also showed the reduced MstX WT had two thiols per monomer, as expected (Additional file 1: Figure S2c). After treating with 3 equivalents (per MstX WT) of H_2_O_2_, only one thiol per monomer was observed. However, MstX:C262S variant showed no thiol per monomer after H_2_O_2_ treatment, indicating that Cys67 was susceptible to oxidation. DTT-treated MstX:C67S proteins contained 0.78 ± 0.2 thiol groups per monomer (Additional file 1: Figure S2c), equal to the thiol content measured in the H_2_O_2_-treated MstX:C67S proteins, indicating Cys262 in MstX:C67S proteins still existed in thiol state.

### MstX:C262S-SSM was specially reduced with electrons from the MSH/Mtr/NADPH pathway

First, we investigated the electron transfer pathway coupled to MstX. To test this, the MSH/Mtr/NADPH electron transfer pathway, an important physiological reducing system in *C. glutamicum*, was used. We employed an in vitro assay system started by adding oxidized MstX:C262S-SSM as substrate for the electron transfer pathway, and the reduction in the absorption at 340 nm due to NADPH consumption was monitored. Only the sample with oxidized MstX:C262S-SSM by linking the MSH/Mtr/NADPH electron transfer pathway showed consumption of NADPH (Additional file 1: Figure S3a). Pedre et al. showed that Trx could reduce a cysteine-MSH mixed disulfide [47]. Thus, we tested whether Trx reduced oxidized MstX:C262S-SSM coupled with TrxR/NADPH pathway. Interestingly, although the reduction ability of Trx for S-mycothiolated MPx (MPx-SSM) linked to TrxR/NADPH pathway was about × 10^2^ in *C. glutamicum* [47], Trx did not show reproducible activity for MstX:C262S-SSM (Additional file 1: Figure S3b). After determining the ability of reduction, we investigated the reduction rate of oxidized MstX:C262S-SSM in a coupled enzyme assay, in which different concentration of oxidized MstX:C262S-SSM was used as substrate linked to *C. glutamicum* MSH/Mtr/NADPH pathway (Additional file 1: Figure S3c). From the Michaelis–Menten kinetic plot, a *k*_cat_ of 0.725 ± 0.014 s^−1^ and a *k*_m_ of 0.811 ± 0.054 μM were obtained, resulting in a specificity constant (*k*_cat_/*k*_m_) of 8.93 × 10^5^ M^−1^ s^−1^.

### MstX had significant thiol transferase (mycoredoxin) and reductase activities but not mycothiol transferase activity

**Fig. 3:**
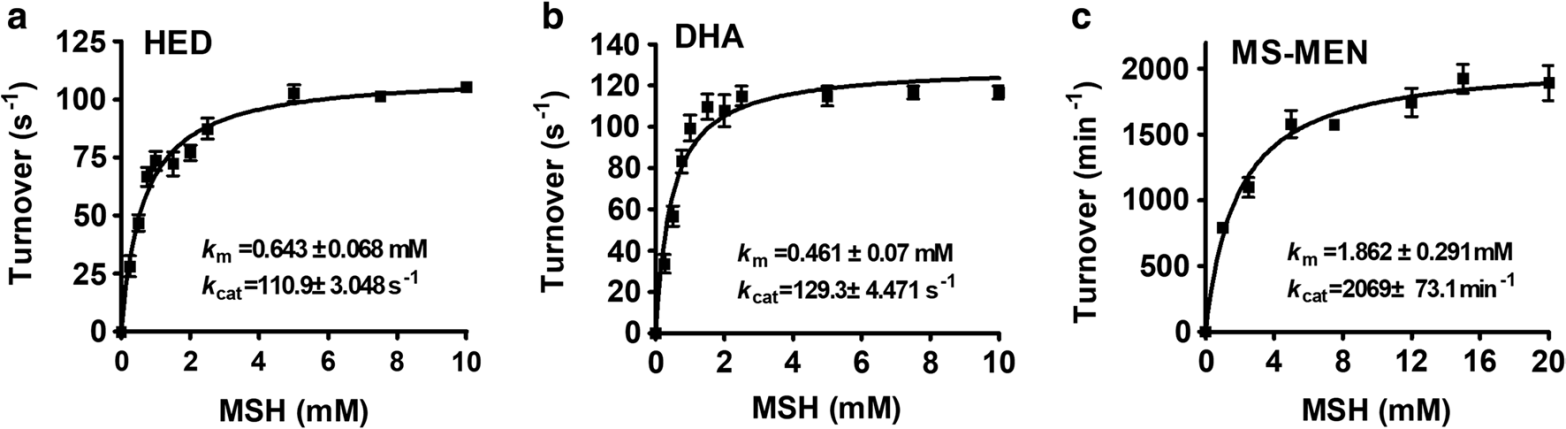
Thiol transferase and reductase activity of MstX proteins as a function of MSH concentration. Thiol transferase and reductase activity of MstX proteins were measured with MSH concentration varying in the range 0–20 mM and saturated concentration of substrates (100 mM HED and DHA, or 50 mM MS-MEN). The Michaelis–Menten plots of MstX activity versus HED (**a**), DHA (**b**), and MS-MEN (**c**) were calculated using the program GraphPad Prism 5. The data were represented as mean ± SD of three independent experiments

According to previous study, GSTX exhibited higher Grx activity [15]. Moreover, Grxs had been known for a long time as GSH-dependent disulfide oxidoreductases that reduced S-glutathiolated mixed disulphides [48]. Thus, the thiol transferase activity of MstX was first determined using HED and MSH as co-substrates in vitro. The kinetic properties of MstX at a fixed concentration of MSH and different concentrations of HED could be measured (Additional file 1: Table S3). The *k*_m_ value, *k*_cat_ value, and catalytic coefficient of HED for MstX were calculated to be 0.192 ± 0.03 mM, 116.73 ± 14 s^−1^, and 6.08 ± 0.18 × 10^5^ M^−1^ s^−1^ (Additional file 1: Table S3). Specific activity of the recombinant MstX proteins as a function of MSH concentration at a fixed concentration of HED was also determined. As shown in Fig. 3a, *k*_m_ value, *k*_cat_ value, and catalytic coefficient of MSH for MstX at 100 mM HED as substrate were 0.643 ± 0.068 mM, 110.9 ± 3.048 s^−1^, and 1.72 ± 0.2 × 10^5^ M^−1^ s^−1^. The above results indicated that MstX had considerably high Mrx activity, similar to that of MSH-dependent reductase (mycoredoxin 1, Mrx1), acting as an oxidoreductase of reducing S-mycothiolated mixed disulphides linked to the MSH electron transfer pathway [49].

MstX proteins also displayed quite high activity of DHA reductase when a fixed concentration of MSH and different concentrations of DHA were used (Additional file 1: Table S3). Moreover, specific activity of the recombinant MstX proteins as a function of MSH concentration at a fixed concentration of DHA was also determined. As shown in Fig. 3b, the *k*_m_ value, *k*_cat_ value, and catalytic coefficient for MSH were 0.461 ± 0.07 mM, 129.3 ± 4.471 s^−1^, 2.8 ± 0.3 × 10^5^ M^−1^ s^−1^, respectively. In addition, we found that MstX displayed slightly higher activity as a DHA reductase than that as HED thiol transferase.

GSTXs were specialized in the reduction of GH. Thus, the recombinant MstX proteins were tested with different concentration of mycothiolyl-menadione (MS-MEN) as substrates to assess its reductase activity. As showed in Additional file 1: Table S4, MstX proteins had mycothiolyl-hydroquinone reductase (MHR) activity with the substrate MS-MEN (*k*_cat_/*k*_m_ around 2.7 ± 0.4 M^−1^ s^−1^) when saturated concentration of MSH was used, in accordance with data obtained on GSTXs from various organisms (YqjG from *E. coli*, 1.8 M^−1^ s^−1^; TvGSTX from *Trametes versicolor*, around 3.7 M^−1^ s^−1^) [8, 15]. Moreover, specific activity of the recombinant MstX proteins as a function of MSH concentration at a fixed concentration of MS-MEN was also determined. As shown in Fig. 3c, the *k*_m_ value, *k*_cat_ value, and catalytic coefficient for MSH were 1.862 ± 0.291 mM, 2069 ± 73.1 min^−1^, 1.9 ± 0.3 M^−1^ s^−1^, respectively, similar to those of TvGSTX3 from the *Trametes versicolor* [15]. Because GSTXs were initially identified as the GST omegas (GSTOs), we investigated if MstX had the ability of reducing MS-PAP, which was similar to glutathionyl-phenylacetophenone (GS-PAP) that was usually reduced by GSTOs [4]. As shown in Additional file 1: Table S4, MstX was inactive toward MS-PAP when MSH was used, indicating that MstX behaved like previously reported GSTXs.

Next, we investigated whether MstX had the ability of conjugating MSH to a variety of exogenous and endogenous electrophilic compounds, which was a typical trait for Ser/Tyr-GSTs class. However, no MSH-transferase activity was detected for the recombinant MstX proteins, when CDNB and monobromobimane (mBBr) were used as substrates (Additional file 1: Table S3). We speculated that the active Cys in MstX could not perform the nucleophilic attack on substrate CDNB and mBBr. MstX therefore had different characteristic from those of most other GSTs, and functionally resembled Grx activity.

### MstX had low activity as MSH peroxidase

**Fig. 4:**
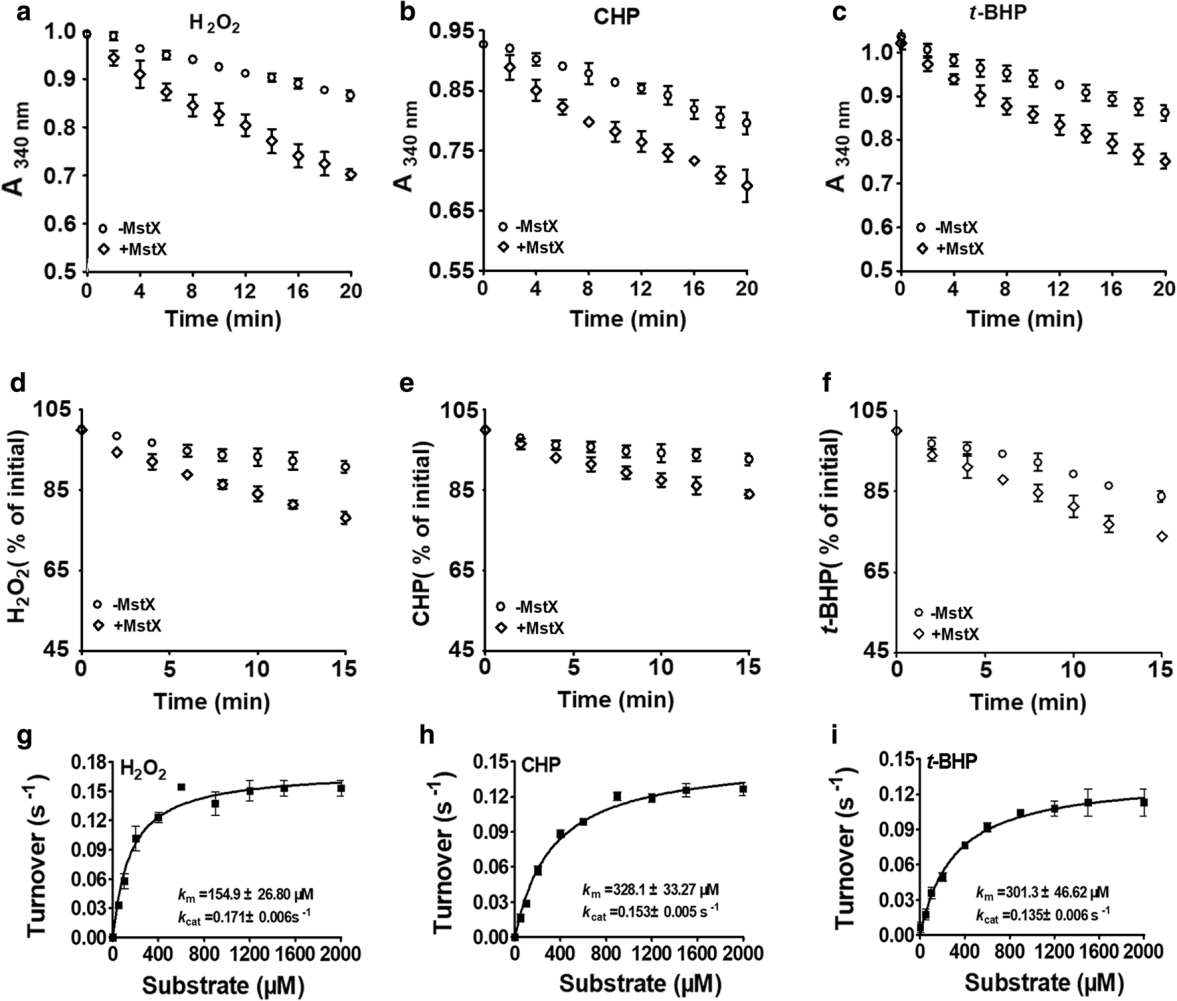
Peroxidase activities of MstX. **a**–**c** The coupled assay of the MstX/MSH/Mtr/NADPH pathway with H_2_O_2_, CHP, and *t*-BHP as substrates. Peroxidase activities were determined by recording NADPH oxidation at 340 nm. A control reaction in the absence of MstX was included. **d**–**f** The FOX assay of the MstX/MSH/Mtr pathway with H_2_O_2_, CHP, and *t*-BHP as substrates. The consumption of H_2_O_2_, CHP, and *t*-BHP by the FOX assay in function of time was shown. **g**–**i** The Michaelis–Menten plots of MstX activity versus H_2_O_2_, CHP, and *t*-BHP were performed with the reaction mixtures described in “Methods”. The data were presented as means of the values obtained from three independent assays. Kinetic parameters were calculated by non-linear regression using the program GraphPad Prism 5

Many members of GSTs family exhibited GSH peroxidase activity against peroxides [50–52]. Moreover, as MstX showed a role in the defence against oxidants (Figs. 1, 2), we then investigated whether MstX proteins were also active as MSH peroxidases. H_2_O_2_, CHP, and *t*-BHP were added to the MSH/Mtr/NADPH electron transfer pathway in the presence and absence of MstX. Addition of MstX to the MSH/Mtr/NADPH system resulted in a slight reduction of the absorption at 340 nm compared to only the MSH/Mtr/NADPH system (Fig. 4a–c). With the FOX assay, we tested the residual peroxide concentration after the reaction. Addition of MstX also caused a slight decrease in H_2_O_2_, CHP, and *t*-BHP content compared to that in only the MSH/Mtr/NADPH system (Fig. 4d–f). The kinetic parameters of MstX were determined with a fixed concentration of MstX (1 μM) and different concentrations of H_2_O_2_, CHP, and *t*-BHP (0–2000 μM). The *k*_m_ value, *k*_cat_ value, and catalytic coefficient of MstX were calculated to be 154.9 ± 26.80 μM, 0.171 ± 0.006 s^−1^, and 11.0 ± 0.6 × 10^2^ M^−1^ s^−1^ for H_2_O_2_; 328.1 ± 33.27 μM, 0.153 ± 0.005 s^−1^, and 4.7 ± 0.2 × 10^2^ M^−1^ s^−1^ for CHP; 301.3 ± 46.62 μM, 0.135 ± 0.006 s^−1^, and 4.5 ± 0.6 × 10^2^ M^−1^ s^−1^ for *t*-BHP (Fig. 4g–i). These results indicated that the peroxide scavenging role of MstX was at a relatively low rate of 10^2^ M^−1^ s^−1^ (using 500 µM MSH), which were several orders of magnitudes lower than the reaction rate of the known peroxidases of *C. glutamicum* (10^5^ to 10^7^ M^−1^ s^−1^) [22–24]. Together, MstX proteins showed low but reproducible activity against these three substrates.

### Cys67 was essential for MstX’s thiol transferase and peroxidase activity

**Fig. 5:**
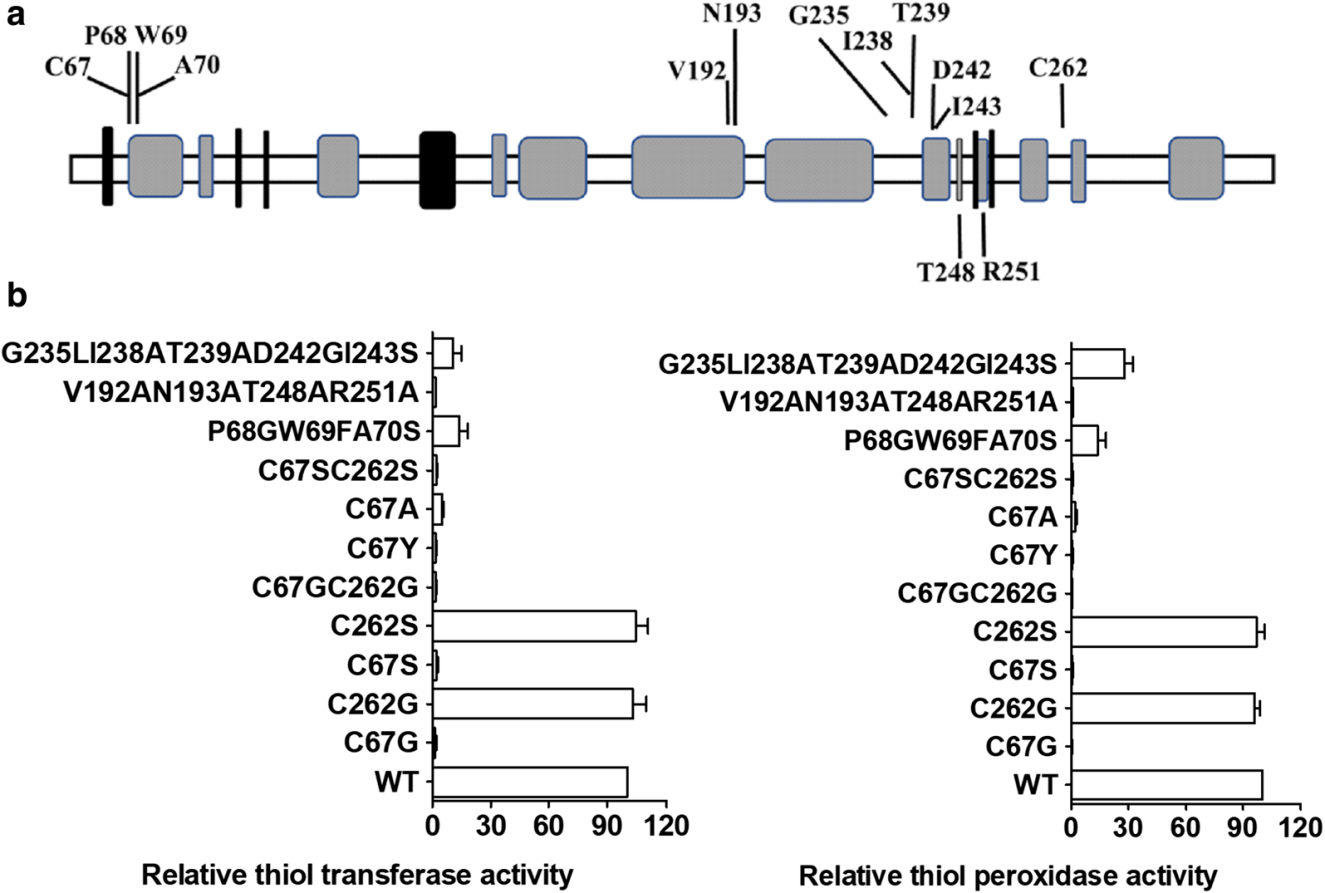
Effect of amino acid change on the mycoredoxin and peroxidase activity of MstX. **a** Secondary-structure prediction for MstX protein. Black boxes corresponded to proposed α-helices and grey boxes to β-strands. The positions of the amino acid residues which were mutated in the present study were shown. **b** Percentage of specific enzyme activity in the HED assay (left panel) and H_2_O_2_ assay (right panel) of the recombinant MstX versions with the indicated amino acid changes, relative to the WT protein. The activities were measured as described in “Additional file 1: Table S3 and Fig. 5”. Values were the means for three independent experiments

The secondary structure prediction showed Cys67 in MstX was located between a *β*-strand and an *α*-helix region in the model of Fig. 5a. Previous studies showed the Cys residue, completely conserved in all reference GSTX, was essential for thiol transferase, indicating Cys67 was required for the activity of MstX as a thiol transferase. Another cysteine residue at position 262 of MstX did not have an equivalent in other reference GSTXs. In order to investigate the role of Cys in function of MstX, we constructed the MstX:C67G, MstX:C67S, MstX:C67A, MstX:C67Y, MstX:C262S, and MstX:C262G variants via site-directed mutation. The activity of these variants towards HED and MSH was measured and the steady-state kinetic constants were compared with that of MstX WT. As shown in Fig. 5b, MstX:C67G, MstX:C67S, MstX:C67A, and MstX:C67Y reduced the thiol transferase activity of MstX to the same undetectable levels as background value from the reactive mixture in the absence of MstX (Fig. 5b). However, the MstX:C262G and MstX:C262S variants, in which Cys67 was left unchanged, had enzyme activity levels comparable with that of the MstX WT, indicating that the thiol transferase activity of MstX was strictly related with a single cysteine residue at position 67, and operated through using a monothiol mechanism.

Next, we tested whether Cys67 and Cys262 of MstX for peroxidase activity also showed the behavior. As shown in Fig. 5b, MstX peroxidase activity depended on the presence of a cysteine residue at position 67, no peroxidase activity was observed in the MstX:C67G, MstX:C67S, MstX:C67A, and MstX:C67Y variants, suggesting involvement of the Cys in the reaction mechanism. However, the MstX:C262G and MstX:C262S variant showed comparable activity with MstX WT.

### The three residues adjacent to Cys67 and C-terminal domain of MstX were also required for the activity

The three residues adjacent to the cysteine at position 67, consisting of the proline, tryptophan, and asparagine (Pro-Trp-Ala, P-W-A), were also completely conserved in all reference GSTXs (Additional file 1: Figure S1). Thus, we tested whether replacing Cys67 plus the adjacent residues Pro68, Trp69 and Ala70 in MstX with the conserved Cys-Gly-Phe-Ser (CGFS) sequence of monothiol Grxs active site had some effect on the activity of the protein. Although CGFS motif could interact with GSH, monothiol Grxs was unable to deglutathionylate the small mixed disulfide between β-mercaptoethanol and a GSH moiety [53]. To test this, MstX: P68GW69FA70S three variant was constructed. The MstX: P68GW69FA70S variant protein with the CGFS motif of Grx5 showed no detectable activity as a thiol transferase with HED (Fig. 5b). This confirmed that, although Cys67 was required for MstX activity, the adjacent residues were also important. Similarly, the three residues adjacent to Cys67 were also very vital for MstX peroxidase activity.

Previous study showed that the C-terminal domain of GST was also required for thiol transferase activity in addition to the thioredoxin fold domain containing C-P-W-A motif [13]. Nine residues (Val192, Asn193, Gly235, Ile238, Thr239, Asp242, Ile243, Thr248, and Arg251) in the C-terminal region of MstX were conserved at equivalent positions of GSTX homologues from other species, belonging to the N-capping box, the hydrophobic staple motif and the hydrophobic co-substrate site, respectively (Additional file 1: Figure S1). Thus, we constructed MstX: V192AN193AT248AR251A and MstX: G235LI238AT239AD242GI243S variants separately and studied their effects on activity. These changes seriously affected the enzyme activity (Fig. 5b), indicating C-terminal domain of MstX were also important for maintaining enzyme activity.

### *mstX* expression

**Fig. 6:**
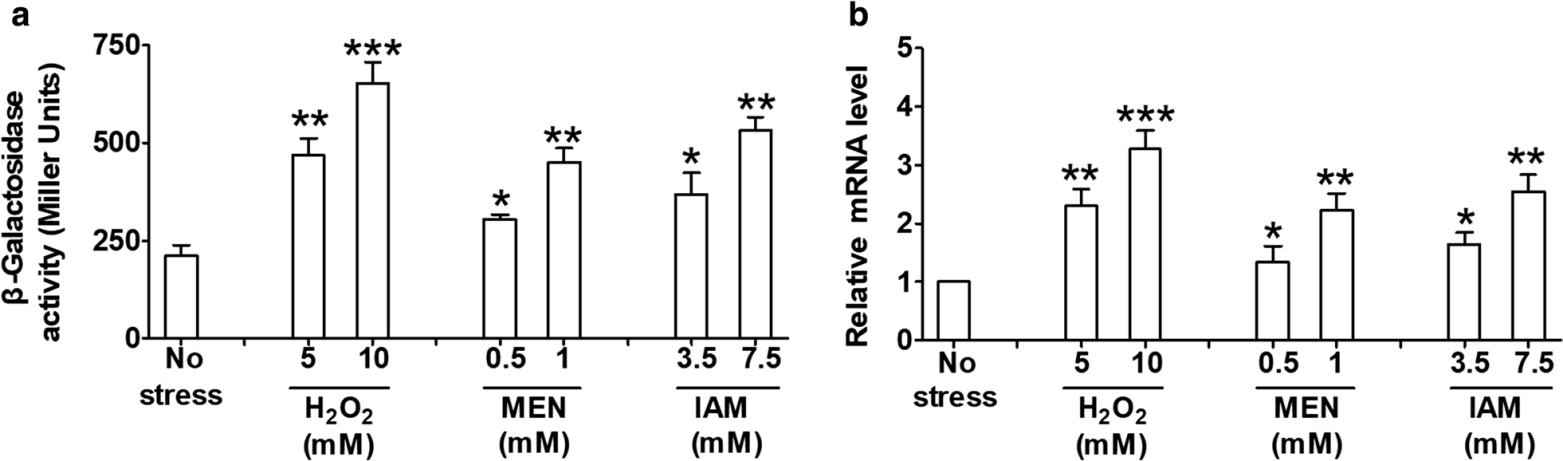
Stress response of *mstX* in *C. glutamicum.* **a** β-Galactosidase analysis of the *mstX* promoter activity was performed using the transcriptional *P*_*mstX*_*::lacZY* chromosomal fusion reporter expressed in *C. glutamicum* RES167 parental strain. 100 μl of exponentially growing *C. glutamicum* cells treated with different toxic agents at indicated concentrations for 30 min was added to the enzyme reaction system. β-Galactosidase activity was assayed as described in “Methods”. Mean values with standard deviations (error bar) from at least three repeats were shown. **b** qRT-PCR assay was performed to analyze the expression of *mstX*. Exponentially growing *C. glutamicum* RES167 parental strains were exposed to different toxic agents at indicated concentrations for 30 min. The levels of *mstX* expression were determined by quantitative RT-PCR. The mRNA levels were presented relative to the value obtained from cells without treatment. The values represent the mean results from three independent cultivations, with standard errors

Since *C. glutamicum mstX* provided resistance to various agents, we performed the *lacZY* activity profiling and qRT-PCR analysis to determine the expression pattern of *mstX* in response to stress conditions. The *lacZY* activity of *P*_*mstX*_*::lacZY* chromosomal promoter fusion reporter in *C. glutamicum* RES167 parental strain was quantitatively measured in bacterial cells either untreated or treated with different agents (Fig. 6a). The level of *mstX* expression was increased by approximately 3.1-, 2.1-, or 2.51-fold in the *C. glutamicum* RES167 parental reporter strain treated with 10 mM H_2_O_2_, 1 mM MEN, or 7.5 mM IAM, respectively, as compared to untreated samples (Fig. 6a). Further, expression of the *P*_*mstX*_*::lacZY* fusion displayed a dose-dependent increase in response to these adverse environmental conditions (Fig. 6a). These results clearly demonstrated that environmental stress induced *mstX* expression, which in turn directly contributed to tolerance of *C. glutamicum* to these stress conditions. A similar pattern of *mstX* expression in response to environmental conditions was also observed by qRT-PCR analysis (Fig. 6b).

## Conclusions

Glutathione transferases (GSTs) from the Xi classes had a catalytic cysteine residue, giving them reductase and thiol transferase activities. GSTX can protect against irreversible oxidation and act as a redox switch regulating metabolic pathways. Here, we presented the biochemical and structural analysis of *C. glutamicum* MSH S-transferase Xi class (MstX). Our data demonstrated that *C. glutamicum* MstX not only had multiple reductase activities, including dehydroascorbate reductase, mycothiolyl-hydroquinone reductase and MSH peroxidase, but also could take over the role of Mrx and reduce the protein-MSH mixed disulfide using a monothiol mechanism, thereby contributing to better understanding of MSH-dependent reactions. However, MstX had no glutathionyl-acetophenones reductase activity so far thought to be specific to GSTOs. In addition, *C. glutamicum* MstX was involved in the cellular defense against various hostile environments. Thus, the experiments reported here first revealed the previously unrecognized contribution of the members of MST family to the demycothionylation of proteins, substrate reduction, and cell resistance. Moreover, these detailed understanding on the bioactive MstX including its biochemical feature and action mechanism might be helpful to establish control strategies during ferment process, where a series of unfavorable circumstances inevitably were generated.

## Supplementary information

**Additional file 1: Table S1.** Bacterial strains and plasmids used in this study. **Table S2.** Primers used in this study. **Table S3.** Activity of recombinant MstX of *C. glutamicum* with CDNB, mBBr, HED, and DHA. **Table S4**. Activity of recombinant MstX of *C. glutamicum* with MS-MEN and MS-PAP. **Figure S1.** Multiple sequences alignment of *C. glutamicum* NCgl1216 with representative Archaea and Gram-positive GST Xi class from *H. lacusprofundi* (YP_002565306), *N. magadii* (YP_003479316), *Str. agalactiae* (NP_687815), and *E. coli YqjG* (NP_417573) and *Saccharomyces cerevisiae* glutathione transferase 2 Omega-like Gto2 (YKR076 W). **Figure S2.** Redox response of MstX in vitro. **Figure S3.** Oxidized MstX:C262S-SSM was mainly reduced via the Mtr/MSH/NADPH pathway.

## Data Availability

All the data generated or analyzed during this study are included in the manuscript and its additional file.

## Abbreviations

ROS: reactive oxygen species
GST: glutathione transferase
GSTX: GST Xi class
GSTO: GST omega class
MstX: mycothiol S-transferase Xi class
GHR: glutathionyl-hydroquinone reductase
GH: glutathionyl-hydroquinone
GSH: glutathione
HED: β-hydroxyethyl disulphide
MstX:C262S-SSM: the mixed disulphide between MSH and MstX:C262S-SH
CDNB: 1-chloro 2,4-dinitrobenzene
DHA: dehydroascorbate
Grxs: glutaredoxins
Trx: thioredoxin
Mtr: mycothione reductase
MSH: mycothiol
MEN: menadione
Mrx: mycoredoxin
CHP: cumene hydroperoxide
LMW: low molecular weight
Gto2: glutathione transferase 2 omega-like

## References

[1] Sheehan D, Meade G, Foley VM, Dowd CA (2001). Structure, function and evolution of glutathione transferases: implications for classification of non-mammalian members of an ancient enzyme superfamily. Biochem J.

[2] Mannervik B (2012). Five decades with glutathione and the GSTome. J Biol Chem.

[3] Hayes JD, Flanagan JU, Jowsey IR (2005). Glutathione transferases. Annu Rev Pharmacol Toxicol.

[4] Lallement PA, Brouwer B, Keech O, Hecker A, Rouhier N (2014). The still mysterious roles of cysteine-containing glutathione transferases in plants. Front Pharmacol..

[5] Schröder P, Scheer CE, Diekmann F, Stampfl A (2007). How plants cope with foreign compounds. Translocation of xenobiotic glutathione conjugates in roots of barley (*Hordeum vulgare*). Environ Sci Pollut Res Int..

[6] Meux E, Prosper P, Ngadin A, Didierjean C, Morel M, Dumarçay S, Lamant T, Jacquot JP, Favier F, Gelhaye E (2011). Glutathione transferases of *Phanerochaete chrysosporium*: a S-glutathionyl-P-hydroquinone reductase belongs to a new structural class. J Biol Chem.

[7] Board PG, Coggan M, Chelvanayagam G, Easteal S, Jermiin LS, Schulte GK, Danley DE, Hoth LR, Griffor MC, Kamath AV, Rosner MH, Chrunyk BA, Perregaux DE, Gabel CA, Geoghegan KF, Pandit J (2000). Identification, characterization, and crystal structure of the Omega class glutathione transferases. J Biol Chem.

[8] Rossjohn J, Polekhina G, Feil SC, Allocati N, Masulli M, Ilio CD, Parker MW (1998). A mixed disulfide bond in bacterial glutathione transferase: functional and evolutionary implications. Structure..

[9] Cromer BA, Morton CJ, Board PG, Parker MW (2002). From glutathione transferase to pore in a CLIC. Eur Biophys J.

[10] Lallement PA, Roret T, Tsan P, Gualberto JM, Girardet JM, Didierjean C, Rouhier N, Hecker A (2016). Insights into ascorbate regeneration in plants: investigating the redox and structural properties of dehydroascorbate reductases from *Populus trichocarpa*. Biochem J..

[11] Mashiyama ST, Malabanan MM, Akiva E, Bhosle R, Branch MC, Hillerich B, Jagessar K, Kim J, Patskovsky Y, Seidel RD, Stead M, Toro R, Vetting MW, Almo SC, Armstrong RN, Babbitt PC (2014). Large-scale determination of sequence, structure, and function relationships in cytosolic glutathione transferases across the biosphere. PLoS Biol..

[12] Green AR, Hayes RP, Xun L, Kang C (2012). Structural understanding of the glutathione-dependent reduction mechanism of glutathionyl-hydroquinone reductases. J Biol Chem.

[13] Garcerá A, Barreto L, Piedrafita L, Tamarit J, Herrero E (2006). *Saccharomyces cerevisiae* cells have three Omega class glutathione S-transferases acting as 1-Cys thiol transferases. Biochem J..

[14] Lallement PA, Meux E, Gualberto JM, Dumarcay S, Favier F, Didierjean C, Saul F, Haouz A, Morel-Rouhier M, Gelhaye E, Rouhier N, Hecker A (2015). Glutathionyl-hydroquinone reductases from poplar are plastidial proteins that deglutathionylate both reduced and oxidized glutathionylated quinones. FEBS Lett.

[15] Schwartz M, Perrot T, Deroy A, Roret T, Morel-Rouhier M, Mulliert G, Gelhaye E, Favier F, Didierjean C (2018). *Trametes versicolor* glutathione transferase Xi 3, a dual Cys-GST with catalytic specificities of both Xi and Omega classes. FEBS Lett.

[16] Frei B, England L, Ames BN (1989). Ascorbate is an outstanding antioxidant in human blood plasma. Proc Natl Acad Sci USA.

[17] Oide S, Gunji W, Moteki Y, Yamamoto S, Suda M, Jojima T, Yukawa H, Inui M (2015). The respiratory chain of *Corynebacterium glutamicum*. Appl Environ Microbiol.

[18] Atichartpongkul S, Loprasert S, Vattanaviboon P, Whangsuk W, Helmann JD, Mongkolsuk S (2001). Bacterial Ohr and OsmC paralogues define two protein families with distinct functions and patterns of expression. Microbiology.

[19] Newton GL, Buchmeier N, Fahey RC (2008). Biosynthesis and functions of mycothiol, the unique protective thiol of *Actinobacteria*. Microbiol Mol Biol Rev.

[20] Liu YB, Long MX, Yin YJ, Si MR, Zhang L, Lu ZQ, Wang Y, Shen XH (2013). Physiological roles of mycothiol in detoxification and tolerance to multiple poisonous chemicals in *Corynebacterium glutamicum*. Arch Microbiol.

[21] Si M, Long M, Chaudhry MT, Xu Y, Zhang P, Zhang L, Shen X (2014). Functional characterization of *Corynebacterium glutamicum* mycothiol S-conjugate amidase. PLoS ONE.

[22] Si M, Xu Y, Wang T, Long M, Ding W, Chen C, Guan X, Liu Y, Wang Y, Shen X, Liu SJ (2015). Functional characterization of a mycothiol peroxidase in *Corynebacterium glutamicum* that uses both mycoredoxin and thioredoxin reducing systems in the response to oxidative stress. Biochem J..

[23] Si M, Zhang L, Chaudhry MT, Ding W, Xu Y, Chen C, Akbar A, Shen X, Liu SJ (2015). *Corynebacterium glutamicum* methionine sulfoxide reductase A uses both mycoredoxin and thioredoxin for regeneration and oxidative stress resistance. Appl Environ Microbiol.

[24] Si M, Wang T, Pan J, Lin J, Chen C, Wei Y, Lu Z, Wei G, Shen X (2017). Graded response of the multifunctional 2-cysteine peroxiredoxin, CgPrx, to increasing levels of hydrogen peroxide in *Corynebacterium glutamicum*. Antioxid Redox Signal.

[25] Newton GL, Leung SS, Wakabayashi JI, Rawat M, Fahey RC (2011). The DinB superfamily includes novel mycothiol, bacilli thiol, and glutathione S-transferases. Biochemistry.

[26] Shen XH, Jiang CY, Huang Y, Liu ZP, Liu SJ (2005). Functional identification of novel genes involved in the glutathione-independent gentisate pathway in *Corynebacterium glutamicum*. Appl Environ Microbiol.

[27] Bussmann M, Baumgart M, Bott M (2010). RosR (Cg1324), a hydrogen peroxide-sensitive MarR-type transcriptional regulator of *Corynebacterium glutamicum*. J Biol Chem.

[28] Si M, Su T, Chen C, Liu J, Gong Z, Che C, Li G, Yang G (2018). OhsR acts as an organic peroxide-sensing transcriptional activator using an S-mycothiolation mechanism in *Corynebacterium glutamicum*. Microb Cell Fact.

[29] Si M, Chen C, Su T, Che C, Yao S, Liang G, Li G, Yang G (2018). CosR is an oxidative stress sensing a MarR-type transcriptional repressor in *Corynebacterium glutamicum*. Biochem J..

[30] Su T, Si M, Zhao Y, Liu Y, Yao S, Che C, Chen C (2018). A thioredoxin-dependent peroxiredoxin Q from *Corynebacterium glutamicum* plays an important role in defense against oxidative stress. PLoS ONE.

[31] Si M, Zhao C, Burkinshaw B, Zhang B, Wei D, Wang Y, Dong TG, Shen X (2017). Manganese scavenging and oxidative stress response mediated by type VI secretion system in *Burkholderia thailandensis*. Proc Natl Acad Sci USA.

[32] Rawat M, Newton GL, Ko M, Martinez GJ, Fahey RC, Av-Gay Y (2002). Mycothiol-deficient *Mycobacterium smegmatis* mutants are hypersensitive to alkylating agents, free radicals, and antibiotics. Antimicrob Agents Chemother.

[33] Ellman GL (1959). Tissue sulfhydryl groups. Arch Biochem Biophys.

[34] Gething MJH, Davidson BE (1976). Molar absorption-coefficient of reduced Ellmans reagent -3-carboxylato-4-nitro-thiophenolate. Eur J Biochem.

[35] Chi BK, Busche T, Van Laer K, Bäsell K, Becher D, Clermont L, Seibold GM, Persicke M, Kalinowski J, Messens J, Antelmann H (2014). Protein S-mycothiolation functions as redox-switch and thiol protection mechanism in *Corynebacterium glutamicum* under hypochlorite stress. Antioxid Redox Signal.

[36] Whitbread AK, Masoumi A, Tetlow N, Schmuck E, Coggan M, Board PG (2005). Characterization of the Omega-class of glutathione transferases. Methods Enzymol.

[37] Holmgren A (1979). Glutathione-dependent synthesis of deoxyribonucleotides. Purification and characterization of glutaredoxin from *Escherichia coli*. J Biol Chem..

[38] Nickerson WJ, Falcone G, Strauss G (1963). Studies on quinone-thioethers. I. Mechanism of formation and properties of thiodione*. Biochemistry..

[39] Habig WH, Pabst MJ, Jakoby WB (1974). Glutathione S-transferases. The first enzymatic step in mercapturic acid formation. J Biol Chem..

[40] Su T, Si M, Zhao Y, Yao S, Che C, Liu Y, Chen C (2019). Function of alkyl hydroperoxidase AhpD in resistance to oxidative stress in *Corynebacterium glutamicum*. J Gen Appl Microbiol..

[41] Wolff SP (1994). Ferrous ion oxidation in presence of ferric ion indicator xylenol orange for measurement of hydroperoxides. Method Enzymol..

[42] Miller JH (1992). A short course in bacterial genetics: a laboratory manual and handbook for *Escherichia coli* and related bacteria.

[43] Luikenhuis S, Perrone G, Dawes IW, Grant CM (1998). The yeast *Saccharomyces cerevisiae* contains two glutaredoxin genes that are required or protection against reactive oxygen species. Mol Biol Cell.

[44] Kohanski MA, Dwyer DJ, Hayete B, Lawrence CA, Collins JJ (2007). A common mechanism of cellular death induced by bactericidal antibiotics. Cell.

[45] Carmel-Harel O, Storz G (2000). Roles of the glutathione- and thioredoxin-dependent reduction systems in the *Escherichia coli* and *Saccharomyces cerevisiae* responses to oxidative stress. Annu Rev Microbiol.

[46] Halliwell B, Gutteridge JM (1984). Oxygen toxicity, oxygen radicals, transition metals and disease. Biochem J..

[47] Pedre B, Van Molle I, Villadangos AF, Wahni K, Vertommen D, Turell L, Erdogan H, Mateos LM, Messens J (2015). The *Corynebacterium glutamicum* mycothiol peroxidase is a reactive oxygen species-scavenging enzyme that shows promiscuity in thiol redox control. Mol Microbiol.

[48] Lillig CH, Berndt C, Holmgren A (2008). Glutaredoxin systems. Biochim Biophys Acta.

[49] Van Laer K, Buts L, Foloppe N, Vertommen D, Van Belle K, Wahni K, Roos G, Nilsson L, Mateos LM, Rawat M, van Nuland NA, Messens J (2012). Mycoredoxin-1 is one of the missing links in the oxidative stress defence mechanism of *Mycobacteria*. Mol Microbiol.

[50] Hurst R, Bao Y, Jemth P, Mannervik B, Williamson G (1998). Phospholipid hydroperoxide glutathione peroxidase activity of human glutathione transferases. Biochem J..

[51] Zhao T, Singhal SS, Piper JT, Cheng J, Pandya U, Clark-Wronski J, Awasthi S, Awasthi YC (1999). The role of human glutathione S transferases hGSTA1-1 and hGSTA2-2 in protection against oxidative stress. Arch Biochem Biophys..

[52] Prabhu KS, Reddy PV, Jones EC, Liken AD, Reddy CC (2004). Characterization of a class alpha glutathione S transferase with glutathione peroxidase activity in human liver microsomes. Arch Biochem Biophys.

[53] Tamarit J, Bell G, Cabisco E, Herrero E, Ros J (2003). Biochemical characterization of yeast mitochondrial Grx5 monothiol glutaredoxin. J Biol Chem.

