# Supplementary material for: Characterization of Xi-class mycothiol S-transferase from Corynebacterium glutamicum and its protective effects in oxidative stress
Source: Microb Cell Fact. 2019 Oct 26;18:182. doi: 10.1186/s12934-019-1232-8 (PMC6815410; doi:10.1186/s12934-019-1232-8)
Supplement: Supplementary file 1 — Additional file 1: Table S1. Bacterial strains and plasmids used in this study. Table S2. Primers used in this study. Table S3. Activity of recombinant MstX of C. glutamicum with CDNB, mBBr, HED, and DHA. Table S4. Activity of recombinant MstX of C. glutamicum with MS-MEN and MS-PAP. Figure S1. Multiple sequences alignment of C. glutamicum NCgl1216 with representative Archaea and Gram-positive GST Xi class from H. lacusprofundi (YP_002565306), N. magadii (YP_003479316), Str. agalactiae (NP_687815), and E. coli YqjG (NP_417573) and Saccharomyces cerevisiae glutathione transferase 2 Omega-like Gto2 (YKR076 W). Figure S2. Redox response of MstX in vitro. Figure S3. Oxidized MstX:C262S-SSM was mainly reduced via the Mtr/MSH/NADPH pathway. [file 12934_2019_1232_MOESM1_ESM.docx]

**Additional Data**

**Characterization of Xi-class mycothiol S-transferase from *Corynebacterium glutamicum* and its protective effects in oxidative stress**

Meiru Si^1#^, Chengchuan Che^1#^, Guanxi Li^1^, Xiaona Li^1^, Zhijin Gong^1^, Jinfeng Liu^1^, Ge Yang^1*^, Can Chen^2*^

^1^ College of Life Sciences, Qufu Normal University, Qufu, Shandong 273165, China;

^2^ College of Life Science and Agronomy, Zhoukou Normal University, Zhoukou，Henan 466001, China

Running title: Xi-class mycothiol S-transferase in *Corynebacterium glutamicum*

**^#^** These authors contributed equally to this work

**^*^** Corresponding authors:

Ge Yang, Can Chen

E-mail: yangge100@126.com ; chenc02@126.com

Tel: 86-13953760056; 86-18736207816

**Table S1. Bacterial strains and plasmids used in this study.**

| **Strains or plasmids** | **Relevant genotype description** | **References** |
| --- | --- | --- |
| **Strains** | | |
| ***Corynebacterium glutamicum*** | | |
| RES167 | Restriction-deficient mutant of ATCC13032, Δ(*cglIM-cglIR-cglIIR*) | [1] |
| Δ*mstX* | *mstX* deleted in RES167 | This study |
| ***E. coli*** | | |
| BL21(DE3) | *E. coli* expression host, *hsdS gal* (*λc*I*ts*857 *ind-l* *Sam7 nin-*5 *lac UV5-*T7 gene 1) | Novagen |
| JM109 | *recA1 supE44 endA1 hsdR17 gyrA96 relA1 thi* Δ(*lac-proAB*)F′(*traD36 proABlacI*^q^ *lacΔZM15*) | Stratagene |
| **Plasmids** | | |
| pK18*mobsacB* | Suicide plasmid carrying *sacB* for selecting double crossover in *C. glutamicum*, KAN^r^ | [2] |
| pK18*mobsacB-*Δ*mstX* | Construct used for in-frame deletion of *mstX* | This study |
| pK18*mobsacB-P_mstX_::lacZY* | *P_mstX_::lacZY* fusion in pK18*mobsacB* | This study |
| pXMJ19 | Shuttle vector (*P_tac_ lacI^q^ pBL1 oriV_C. glutamicum_* pK18 *oriV_E. coli_*) | [3] |
| pXMJ19-His_6_ |  | [4] |
| pXMJ19-*mstX* | *mstX* cloned into pXMJ19 for complementation | This study |
| pXMJ19-*mstX:C67S* | *mstX:C67S* cloned into pXMJ19 for complementation | This study |
| pET28a | Expression vector with N-terminal hexahistidine affinity tag | Novagen |
| pET28a*-trx* | *trx* in pET28a | [5] |
| pET28a*-trxR* | *trxR* in pET28a | [5] |
| pET28a*-mstX* | *mstX* in pET28a | This study |
| pET28a*-mstX:C67S* | *mstX:C67S* in pET28a | This study |
| pET28a-*mstX:C67G* | *mstX:C67G* in pET28a | This study |
| pET28a-*mstX:C67A* | *mstX:C67A* in pET28a | This study |
| pET28a-*mstX:C67Y* | *mstX:C67Y* in pET28a | This study |
| pET28a*-mstX:P68G* | *mstX:P68G* in pET28a | This study |
| pET28a-*mstX:W69F* | *mstX:W69F* in pET28a | This study |
| pET28a-*mstX:A70S* | *mstX:A70S* in pET28a | This study |
| pET28a-*mstX:C262S* | *mstX:C262S* in pET28a | This study |
| pET28a-*mstX:C262G* | *mstX:C262G* in pET28a | This study |
| pET28a-*mstX:V192A* | *mstX:V192A* in pET28a | This study |
| pET28a-*mstX:N193A* | *mstX:N193A* in pET28a | This study |
| pET28a-*mstX:G235L* | *mstX:G235L* in pET28a | This study |
| pET28a-*mstX:I238A* | *mstX:I238A* in pET28a | This study |
| pET28a-*mstX:T239A* | *mstX:T239A* in pET28a | This study |
| pET28a-*mstX:D242G* | *mstX:D242G* in pET28a | This study |
| pET28a-*mstX:I243S* | *mstX:I243S* in pET28a | This study |
| pET28a-*mstX:T248A* | *mstX:T248A* in pET28a | This study |
| pET28a-*mstX:R251A* | *mstX:R251A* in pET28a | This study |
| pET28a*-mtr* | *mtr* in pET28a | [4] |

**Additional References**

1. Tauch A, Kirchner O, Löffler B, Götker S, Pühler A, Kalinowski J. Efficient electrotransformation of corynebacterium diphtheriae with a mini-replicon derived from the *Corynebacterium glutamicum* plasmid pGA1. [Curr Microbiol](http://www.ncbi.nlm.nih.gov/pubmed/?term=Efficient+electrotransformation+of+corynebacterium+diphtheriae+with+a+mini-replicon+derived+from+the+Corynebacterium+glutamicum+plasmid+pGA1.). 2002; 45, 362-367.

2. Jakoby M, Ngouoto-Nkili CE, Burkovski A. Construction and application of new *Corynebacterium glutamicum* vectors. Biotechnol Tech.1999; 13, 437-441.

3. K arimova G, Pidoux J, Ullmann A, Ladant D. A bacterial two-hybrid system based on a reconstituted signal transduction pathway. [Proc Natl Acad Sci U S A](http://www.ncbi.nlm.nih.gov/pubmed/9576956).1998; 95, 5752-5756.

4. Si M, Su T, Chen C, Liu J, Gong Z, Che C, Li G, Yang G. OhsR acts as an organic peroxide-sensing transcriptional activator using an S-mycothiolation mechanism in *Corynebacterium glutamicum*. Microb Cell Fact. 2018; 17(1), 200.

5. Su T, Si M, Zhao Y, Liu Y, Yao S, Che C, Chen C. A thioredoxin-dependent peroxiredoxin Q from *Corynebacterium glutamicum* plays an important role in defense against oxidative stress. PLoS One.2018;13(2*)*, e0192674.

**Table S2. Primers used in this study.**

| **Primiers** | **5’-3’ sequence** |  |
| --- | --- | --- |
| CmstX-F | CGCGGATCC AAAGGAGGACAACCCGACTCTAGAGGATCCAAAGGAGGACAACCGTGGCTAACACGTCATCCGA (*Bam*HI) | For cloning *mstX* wild type and variants into pXMJ19 |
| CmstX-R | CAAGAATTC CAGCCAAGCTGAATTCTTACTTCTGAAAAGGTTCAGGG (*Eco*RI) |  |
| OmstX-F | CGCGGATCCGTGGCTAACACGTCATCCGATTGG (*Bam*HI) | For cloning *mstX* wild type and variants into pET28a |
| OmstX-R | CCCAAGCTTTTACTTCTGAAAAGGTTCAGGG (*Hin*dIII) |  |
| DmstX-F1 | CAAGAATTC CTATGACATGATTACGAATTCTGGTGGAAGCCAGGTTGC(*Eco*RI) | To generate pK18*mobsacB-*Δ*mstX* |
| DmstX-R1 | GCAGAGTAACCGAACTCGCCGTCTGCTG |  |
| DmstX-F2 | GGCGAGTTCGGTTACTCTGCCTGGCCCAATTC |  |
| DmstX*-*R2 | CGCGGATCC CAGGTCGACTCTAGAGGATCCCACGTGGGTCGAGTGTTGG(*Bam*HI) |  |
| mstX-C67S-F | CGCTGTTGTTGGC*A*GCGCTTCTTTGTC | To generate *mstX:C67S* DNA fragment |
| mstX-C67S-R | GACAAAGAAGCGC*T*GCCAACAACAGCG |  |
| mstX-C67G-F | AATTGATCGCCTG*A*GTTCTGTGAAGCG | To generate *mstX:C67G* DNA fragment |
| mstX-C67G-R | CGCTTCACAGAAC*T*CAGGCGATCAATT |  |
| mstX-C67A-F | GCTGCCCGCGCA*GC*TCCATGGGCACA | To generate *mstX:C67A* DNA fragment |
| mstX-C67A-R | TGTGCCCATGGA*GC*TGCGCGGGCAGC |  |
| mstX-C67Y-F | CTGCCCGCGCAT*A*TCCATGGGCACA | To generate *mstX:C67Y* DNA fragment |
| mstX-C67Y-R | TGTGCCCATGGA*T*ATGCGCGGGCAG |  |
| mstX-P68G-F | GCCCGCGCATGT*GG*ATGGGCACACCG | To generate *mstX:P68G* DNA fragment |
| mstX-P68G-R | CGGTGTGCCCAT*CC*ACATGCGCGGGC |  |
| mstX-W69F-F | GCGCATGTCCAT*TC*GCACACCGCACT | To generate *mstX:W69F* DNA fragment |
| mstX-W69F-R | AGTGCGGTGTGC*GA*ATGGACATGCGC |  |
| mstX-A70S-F | GCATGTCCATGG*T*CACACCGCACTG | To generate *mstX:A70S* DNA fragment |
| mstX-A70S-R | CAGTGCGGTGTG*A*CCATGGACATGC |  |
| mstX- C262S-F | GACACTTCAAGT*C*TGGCCGCAACAA | To generate *mstX:C262S* DNA fragment |
| mstX- C262S-R | TTGTTGCGGCCA*G*ACTTGAAGTGTC |  |
| mstX- C262G-F | GGACACTTCAAG*G*GTGGCCGCAACA | To generate *mstX:C262G* DNA fragment |
| mstX- C262G-R | TGTTGCGGCCAC*C*CTTGAAGTGTCC |  |
| mstX- V192A-F | CTTCACTGAGG*C*CAACAACGGCG | To generate *mstX:V192A* DNA fragment |
| mstX- V192A-R | CGCCGTTGTTG*G*CCTCAGTGAAG |  |
| mstX- N193A-F | TTCACTGAGGTC*GC*CAACGGCGTATA | To generate *mstX:N193A* DNA fragment |
| mstX- N193A-R | TATACGCCGTTG*GC*GACCTCAGTGAA |  |
| mstX- G235L-F | CGTTACCTCATG*CU*GGATCACATCAC | To generate *mstX:G235L* DNA fragment |
| mstX - G235L-R | GTGATGTGATCC*AG*CATGAGGTAACG |  |
| mstX - I238A-F | CATGGGGGATCAC*GC*CACCGAGGCGGAT | To generate *mstX:I238A* DNA fragment |
| mstX - I238A-R | ATCCGCCTCGGTG*GC*GTGATCCCCCATG |  |
| mstX - T239A-F | GGGGATCACATC*GT*CGAGGCGGATAT | To generate *mstX:T239A* DNA fragment |
| mstX - T239A-R | ATATCCGCCTCG*AC*GATGTGATCCCC |  |
| mstX - D242G-F | CACCGAGGCGG*C*TATCCGCCTCTA | To generate *mstX:D242G* DNA fragment |
| mstX - D242G-R | TAGAGGCGGATA*G*CCGCCTCGGTG |  |
| mstX - I243S-F | CCGAGGCGGATA*G*CCGCCTCTACCC | To generate *mstX:I243S* DNA fragment |
| mstX - I243S-R | GGGTAGAGGCGG*C*TATCCGCCTCGG |  |
| mstX - T248A-F | CGCCTCTACCCA*G*CCCTCGTGCGTT | To generate *mstX:T248A* DNA fragment |
| mstX - T248A-R | AACGCACGAGGG*C*TGGGTAGAGGCG |  |
| mstX - R251A-F | CCAACCCTCGTG*GC*TTTCGATGCCGT | To generate *mstX:R251A* DNA fragment |
| mstX - R251A-R | ACGGCATCGAAA*GC*CACGAGGGTTGG |  |
| P*_mstX_*-F1 | TCCCCCGGGTTTAACTTTTTTGGTTGACCTG(*Sma*I) | To generate pK18*mobsacB-P _mstX_::lacZY* and the 200 bp *mstX* promoter probe |
| P*_mstX_*-R1 | ACTAGTCGTGTTAGCCACTGTGTGGTGTCC(*Spe*I) |  |
| LacZY-F1 | GGACACCACACAGTGGCTAACACGACTAGT CGTGTTAGCCACTGTGTGGTGTCC(*Spe*I) |  |
| LacZY-R | CGCGGATCCTTAAGCGACTTCATTCACCTG(*Bam*HI) |  |

Underlined sites indicate restriction enzyme cutting sites added for cloning. Letters in italic denote the mutation sites in overlap PCR for site-directed mutation.

**Table S3 Activity of recombinant MstX of *C. glutamicum* with CDNB, mBBr, HED, and DHA**

| Substrates |  | MstX | | |
| --- | --- | --- | --- | --- |
|  |  | *k*_m_ (mM) | *k*_cat_ (s^-1^) | *k*_cat_/*k*_m_  ×10^5^ (M^-1^ s^-1^) |
| Mycothiol transferase activity |  |  |  |  |
| CDNB |  | ND | ND | ND |
| mBBr |  | ND | ND | ND |
| Thiol transferase |  |  |  |  |
| HED with MSH |  | 0.192±0.03 | 116.73±14 | 6.08±0.18 |
| DHA reductase |  |  |  |  |
| DHA with MSH |  | 1.115±0.252 | 573.16±44.3 | 5.71±4.1 |

Activity assays were performed as described in experimental procedures with 1 μM MstX using a concentration range of 0.05–10 mM substrates in the presence of 100 mM MSH and 5 μM Mtr. The data were presented as means of values obtained from three independent assays and analyzed by non-linear regression using the program GraphPad Prism 5. ND, not detectable under the conditions used. CDNB, 1-chloro-2, 4-dinitrobenzene. mBBr, monobromobimane.

**Table S4 Activity of recombinant MstX of *C. glutamicum* with MS-MEN and** MS-PAP

| Substrates |  | MSH/Mtr | | |
| --- | --- | --- | --- | --- |
|  |  | *k*_m_ (mM) | *k*_cat_ (min^-1^) | *k*_cat_/*k*_m_  (M^-1^ s^-1^) |
| MS-MEN |  | 2.89±0.5 | 4653±671 | 2.7±0.4 |
| MS-PAP |  | ND | ND | ND |

Activity assays were performed as described in Experimental procedures with 1 μM MstX using a concentration range of substrates (0.05-10 mM MS-PAP, or 0.5-10 mM MS-MEN) in the presence of 100 mM MSH and 5 μM Mtr. Data were represented as mean ± S.D. (n = 3). ND: not detected. MS-MEN: mycothiolyl-menadione, MS-PAP: mycothiolyl-phenylacetophenone.


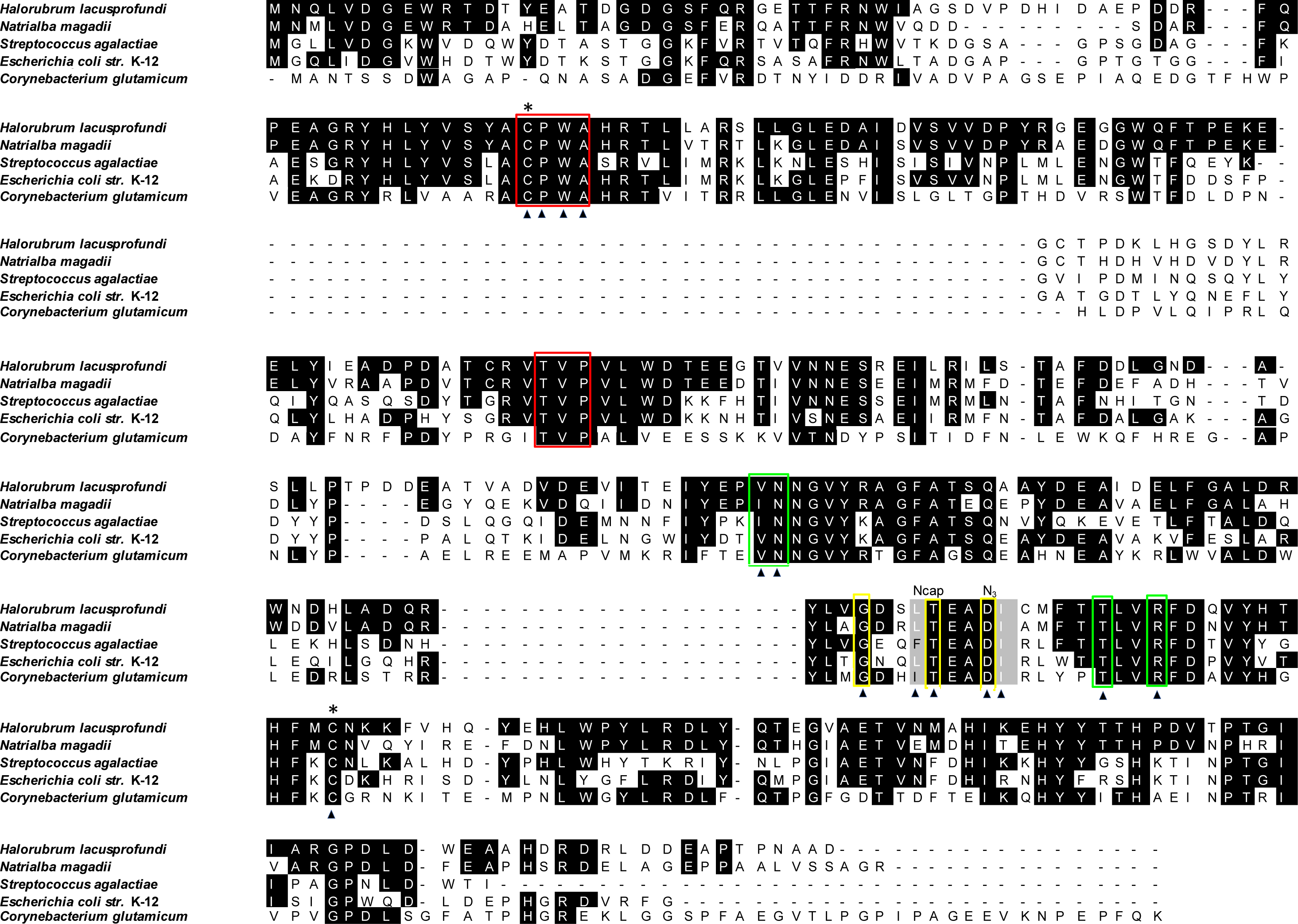


**Figure S1** **Multiple sequences alignment of *C. glutamicum* NCgl1216 with representative Archaea and Gram-positive Xi class GST from *H. lacusprofundi* (YP_002565306), *N. magadii* (YP_003479316), *Str. agalactiae* (NP_687815), and *E. coli YqjG* (NP_417573).** Cys residues were highlighted in black stars. Cysteine and its adjacent residues as well as the residues involved in the GSH-binding were shown in red boxes. The N-capping box residues and the glycine residue were shown in yellow boxes. The hydrophobic staple motif residues were shown in gray. The hydrophobic co-substrate (H-site) was shown in green boxes. All conserved residues were shown in black. The residues mutated were indicated with soild triangle.


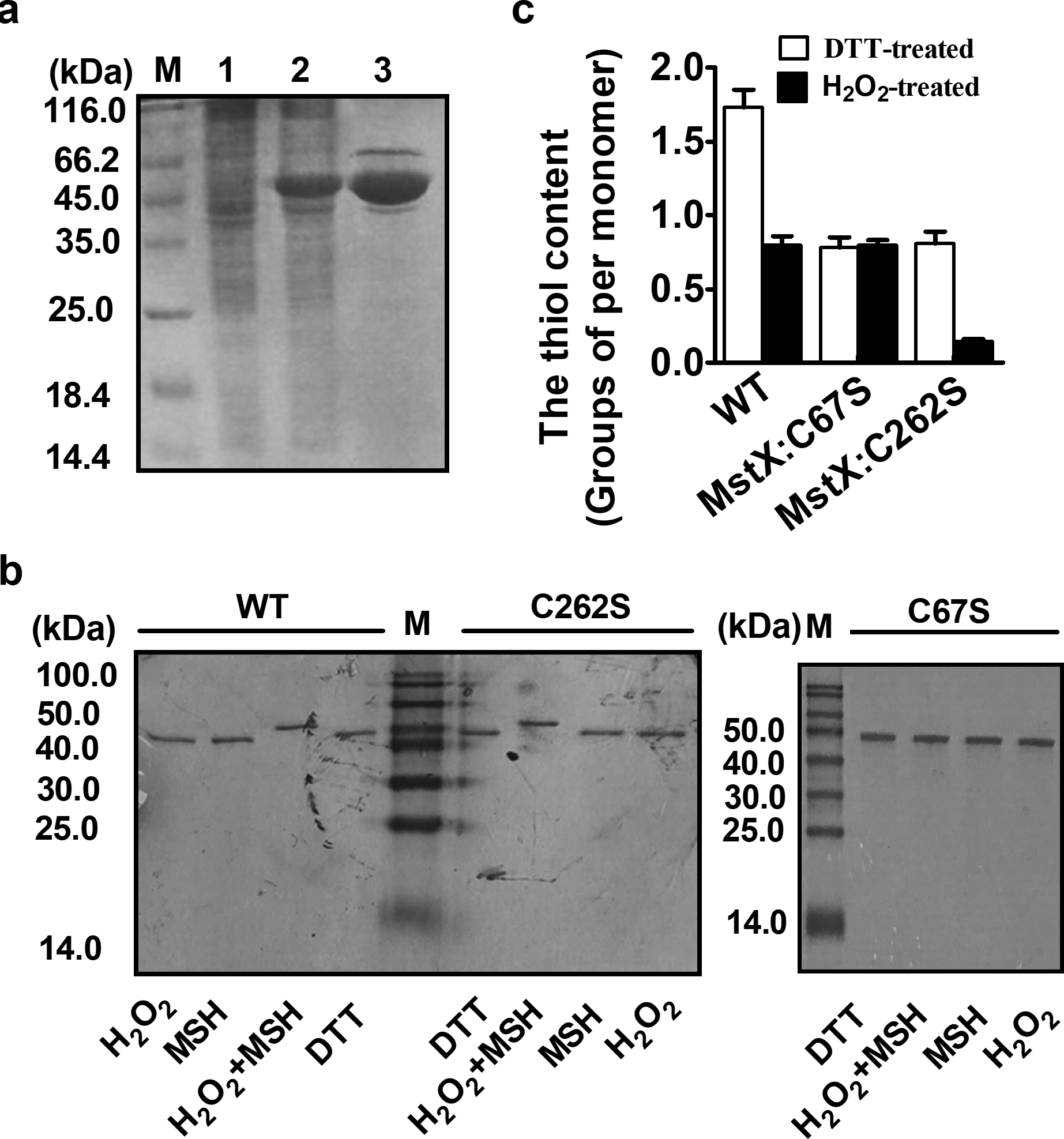


**Figure S2 Redox response of MstX *in vitro*. a Nonreducing SDS-PAGE analysis of proteins expressed in *E. coli* containing pET28a-*mstX* plasmid.** M, broad-range protein marker; lane 1, crude extract (5 μg) without IPTG induction; lane 2, crude extract (5 μg) with induction; lane 3 purified His_6_-MstX protein. **b** Redox response of MstX detected by nonreducing SDS-PAGE. Proteins treated with 50 mM DTT were further incubated with or without H_2_O_2_, MSH, or H_2_O_2_ and MSH, and samples were then separated by 15% nonreducing SDS-PAGE. **c** Quantification of free MstX thiol levels in reduced and oxidized proteins. H_2_O_2_- and DTT-treated proteins were mixed 2 mM with DTNB in 50 mM Tris-HCl buffer (pH 8.0), and the absorbance was monitored at 412 nm against a 2 mM DTNB solution as reference. These data were means of the values obtained from three independent assays.


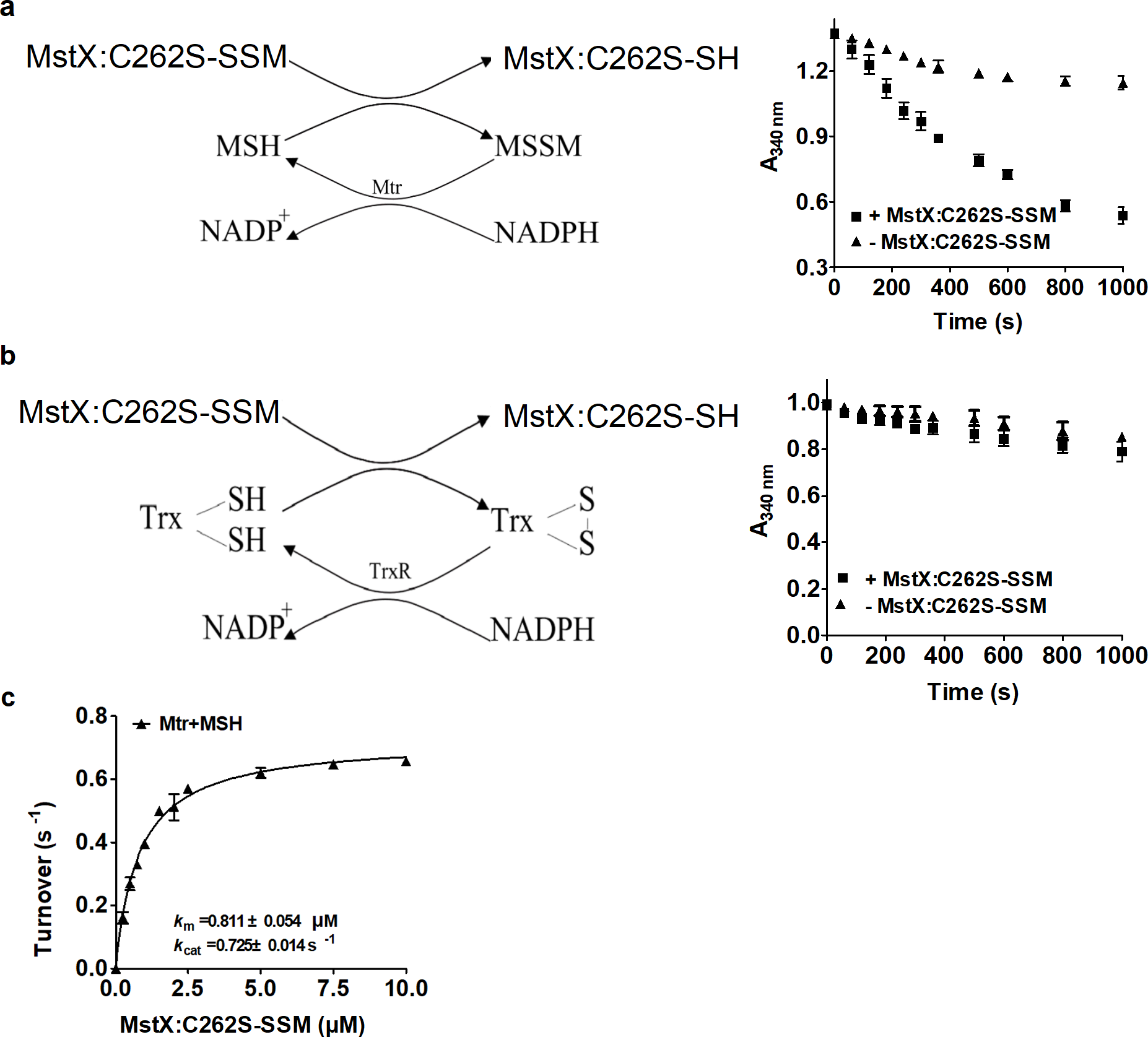


**Figure S3 Oxidized MstX:C262S-SSM was mainly reduced via the Mtr/MSH/NADPH pathway. a and b** Oxidized MstX:C262S-SSM was added as substrate to the MSH/Mtr/NADPH pathway (**a**) and the Trx/TrxR pathway (**b**). A control reaction in the absence of oxidized MstX:C262S-SSM was included. The consumption of NADPH at 340 nm was shown. **c** The reduction of the oxidized MstX:C262S-SSM by the MSH/Mtr/NADPH pathway was evaluated via Michaelis–Menten steady-state kinetics. Different concentrations of oxidized MstX:C262S-SSM were mixed with a pre-incubated mixture of the MSH, Mtr, and NADPH. The decrease in *A*_340 nm_, due to NADPH oxidation, was monitored in function of time.
